# Epigenetics of Muscle- and Brain-Specific Expression of KLHL Family Genes

**DOI:** 10.3390/ijms21218394

**Published:** 2020-11-09

**Authors:** Kenneth C. Ehrlich, Carl Baribault, Melanie Ehrlich

**Affiliations:** 1 Center for Biomedical Informatics and Genomics, Tulane University Health Sciences Center, New Orleans, LA 70112, USA; kehrlich@tulane.edu; 2 Center for Research and Scientific Computing (CRSC), Tulane University Information Technology, Tulane University, New Orleans, LA 70112, USA; cbaribault@tulane.edu; 3 Center for Biomedical Informatics and Genomics, Tulane Cancer Center, Hayward Genetics Program, Tulane University Health Sciences Center, New Orleans, LA 70112, USA

**Keywords:** DNA methylation, chromatin structure, enhancers, super-enhancers, promoters, retrogene, DNaseI-hypersensitive sites, skeletal muscle, brain, topologically associating domains

## Abstract

*KLHL* and the related *KBTBD* genes encode components of the Cullin-E3 ubiquitin ligase complex and typically target tissue-specific proteins for degradation, thereby affecting differentiation, homeostasis, metabolism, cell signaling, and the oxidative stress response. Despite their importance in cell function and disease (especially, *KLHL40*, *KLHL41*, *KBTBD13*, *KEAP1*, and *ENC1*), previous studies of epigenetic factors that affect transcription were predominantly limited to promoter DNA methylation. Using diverse tissue and cell culture whole-genome profiles, we examined 17 *KLHL* or *KBTBD* genes preferentially expressed in skeletal muscle or brain to identify tissue-specific enhancer and promoter chromatin, open chromatin (DNaseI hypersensitivity), and DNA hypomethylation. Sixteen of the 17 genes displayed muscle- or brain-specific enhancer chromatin in their gene bodies, and most exhibited specific intergenic enhancer chromatin as well. Seven genes were embedded in super-enhancers (particularly strong, tissue-specific clusters of enhancers). The enhancer chromatin regions typically displayed foci of DNA hypomethylation at peaks of open chromatin. In addition, we found evidence for an intragenic enhancer in one gene upregulating expression of its neighboring gene, specifically for *KLHL40*/*HHATL* and *KLHL38/FBXO32* gene pairs. Many *KLHL/KBTBD* genes had tissue-specific promoter chromatin at their 5′ ends, but surprisingly, two (*KBTBD11* and *KLHL31*) had constitutively unmethylated promoter chromatin in their 3′ exons that overlaps a retrotransposed *KLHL* gene. Our findings demonstrate the importance of expanding epigenetic analyses beyond the 5′ ends of genes in studies of normal and abnormal gene regulation.

## 1. Introduction

Establishing and maintaining tissue-specific levels of active proteins that help mediate differentiation depends on regulating protein stability as well as transcription, mRNA stability, translation, and post-translational protein modification [1,2,3,4]. Kelch-like (*KLHL*) genes encode substrate-specific adapters for cullin E3 ubiquitination that play a major role in targeting specific proteins for degradation and thus affect differentiation and homeostasis [5,6]. KLHL proteins contain five or six Kelch domains, which mediate substrate recruitment; a BTB/POZ domain, which binds to the Cullin E3 ligase; and a BACK domain with uncertain function that links the other two domains. Like the KLHL proteins, KBTBD proteins have Kelch and BTB/POZ domains and, in some proteins, a BACK domain. They also usually function in specific ubiquitin-mediated proteolysis. Both types of proteins influence fundamental cellular processes including cell signaling, transcription, cell division, cytoskeletal remodeling, response to oxidative stress, apoptosis, and autophagy [6,7]. As a result of the structural and functional similarities of *KLHL* and *KBTBD* genes, we refer to both as being in the *KLHL* gene family.

Consistent with their wide-ranging roles, some of the KLHL or KBTBD proteins are associated with somatic diseases or linked to hereditary disease [6,7]. Downregulation of *KEAP1* (*KLHL19*), the most well-studied *KLHL* family gene, is implicated in many types of cancer [6,8], and cancer-associated mutation of other *KLHL* family genes has been described [5]. Among the other diseases in which *KLHL* family proteins are implicated are myopathies [9,10], diabetes [11], pseudohypoaldosteronism (a mineral transport disorder) [12], and cardiac dysfunction [13]. Although *KLHL* family genes are usually highly tissue-specific in their transcription, almost one-third of the proteins encoded by the 51 genes in this family (including *KBTBD* genes) are poorly understood as to their biological function(s).

Despite the biological importance of the *KLHL* family genes, there has been only a limited examination of their epigenetics, mostly with respect to methylation of the promoter regions, which is usually defined as a region 0.1 to 2 kb upstream of the transcription start site (TSS) and extending up to 1 kb downstream of the transcription start site (TSS) [6]. Most human promoter regions have low or negligible levels of DNA methylation in all tissues and are enriched in CpGs, unlike most of the rest of the genome. Such a region is called a CpG island (CGI) [14]. When a CGI overlapping an active promoter becomes highly methylated, the promoter is almost always silenced. However, the lack of DNA methylation at a promoter and an open chromatin configuration can be seen in many genes that are poised for transcription as well as at those that are actively transcribed.

Enhancers are highly important in establishing tissue-specific expression [4,15]. Enhancer chromatin in genome-wide studies is usually defined as regions enriched in histone H3 lysine 27 acetylation (H3K27ac) and histone H3K4 monomethylation (H3K4me1) while active promoters exhibit H3K27ac enrichment and H3K4 trimethylation (H3K4me3) instead of H3K4me1 and are usually adjacent to the TSS. Enhancers can be immediately upstream of promoters but are frequently found elsewhere [4,16]. Just as tissue-specific or disease-associated DNA methylation can down-regulate promoters, it can also down-modulate enhancers, mostly by limiting the accessibility and/or binding of transcription factors to the enhancer [15,17]. Enhancers are sometimes very far from the promoter that they upregulate. However, they should be in the same higher-order chromatin loop or topologically associating domain (TAD), which usually requires the binding of the CCCTC-binding factor (CTCF) at the ends of the loop [18].

In the current study, we used publicly available transcription and epigenetic profiles of the 17 *KLHL* family genes that show preferential expression in skeletal muscle (SkM) or brain to compare these genes’ transcriptomics and epigenomics in many human tissues and primary cell cultures. Comparisons of chromatin states (enhancer, promoter, and repressed), histone H3 acetylation signal, DNaseI-hypersensitive sites, and DNA methylation profiles between different tissue or cell cultures showed that all four parameters were non-redundantly helpful in understanding the tissue-specific profiles of expression of these genes. In addition, a neuronal cell fraction from brain revealed strong DNA hypomethylated regions that were not apparent in whole brain for several of the brain-associated *KLHL* family genes. As a result of the importance of gene neighborhoods to transcription regulation, we extended our analysis to include genes that surround the *KLHL* family genes and obtained new insights into enhancers from one gene being associated with expression from their gene neighbor. Our analysis reinforces the importance of studying the epigenetics of transcription regulatory regions beyond the canonical upstream promoter region. We show that this allows us to better understand the regulation of tissue-specific expression of *KLHL* family genes.

## 2. Results

### 2.1. Many KLHL and KBTBD Genes Are Expressed Preferentially in SkM or Brain

The three tissues with the most frequent tissue-specific expression of the 51 *KLHL* family genes (with the designation “*KLHL*” or “*KBTBD*”), were SkM, brain, and testis (Supplementary Tables S1 and S2) as determined from the median transcripts per kilobase million (TPM) from hundreds of biological replicates per tissue in the GTEx database [19]. We studied the epigenetics of only SkM- or brain-associated genes because of the availability of many epigenetic profiles for SkM and brain but not for testis. There were ten *KLHL* family genes preferentially expressed in SkM and six in most examined subregions of the brain (Table 1 and Table 2, respectively) using as the definition of preferential expression that the median TPM for SkM (psoas) or brain (frontal cortex) is at least 3-fold higher than the median TPM of non-SkM or non-brain tissue types in the GTEx database, which has 52 tissue types. Five genes (*KBTBD6*, *KBTBD12*, *KLHL1*, *KLHL3*, and *KLHL22*) that were preferentially expressed specifically in cerebellum, the most transcriptionally distinct brain subregion [20], were not included in our study because of the lack of available chromatin state segmentation profiles for this brain subregion. Genes that were preferentially expressed in frontal cortex usually also were in the other examined brain regions, with cerebellum being the most frequent exception (Table 2 and Supplemental Table S2). *KEAP1* (which had its highest expression in SkM (Supplementary Table S2) is by far the most studied of all the *KLHL* family genes and is involved in various diseases [21]; therefore, it is included in our study even though its SkM/non-SkM expression ratio was only 2.4 instead of >3.

### 2.2. Extensive Intragenic Promoter Chromatin and Overlapping DNA Hypomethylation Correlates with the Extremely High Expression of KLHL41 in Skeletal Muscle

**Figure 1:**
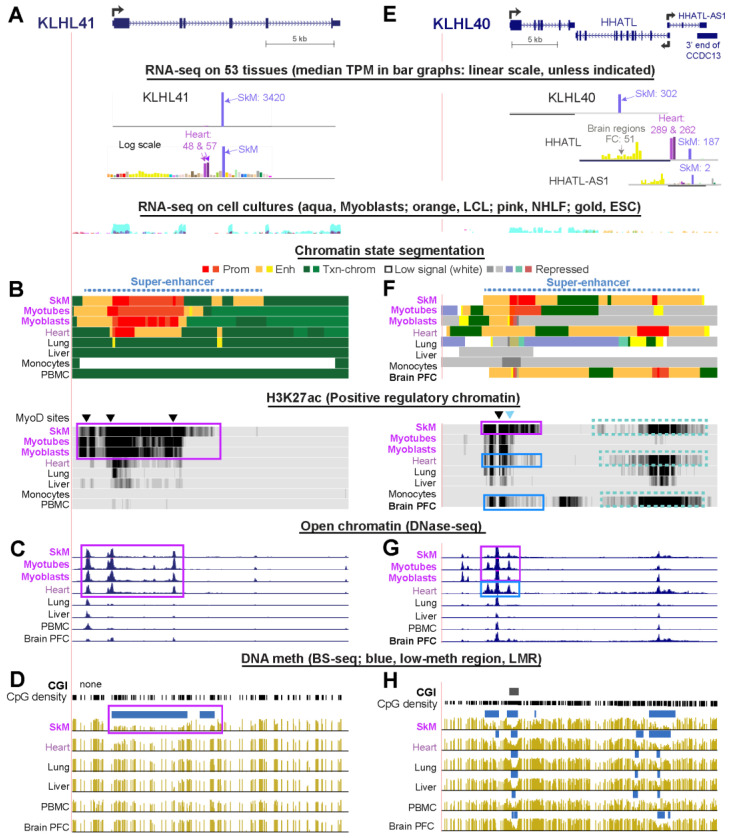
*KLHL40* and *KLHL41*, genes involved in nemaline myopathy, have tissue-specific chromatin and DNA methylation profiles that correlate with their skeletal muscle-specific expression. (**A**–**D**) *KLHL41*, chr2:170,363,256-170,383,339 and (**E**–**H**) *KLHL40* and neighboring genes *HHATL* and the 3′-end of *CCDC13*, chr3:42,719,627-42,749,414. (**A**,**E**) RNA-seq profiles for tissues, either linear or log_10_-transformed TPM bar graphs with the black horizontal indicating the region used for determining TPM; RNA-seq for cell cultures, overlay signal for the indicated four cell cultures. (**B**,**F**) Chromatin state segmentation [4] is based on key histone methylation and acetylation profiles (see Methods). Dotted blue line over the chromatin state tracks, SkM super-enhancer; H3K27ac signal, vertical viewing ranges 0–20 and 0–10 for *KLHL41* and *KLHL40*, respectively; (**C**,**G**) DNaseI hypersensitivity, vertical viewing range 0–20; (**D**,**H**) BS-seq, bisulfite-seq; blue bars, regions of significantly lower methylation than in the whole the genome for the same tissue. SkM, skeletal muscle (psoas), brain PFC, pre-frontal cortex; heart, left ventricle. All tracks are horizontally aligned in this and other figures and are from the UCSC Genome Browser (http://www.genome.ucsc.edu/) hg19 reference genome. Purple boxes in (**B**,**D**), epigenetic marks seen at *KLHL41* specifically in the *KLHL41*-expressing SkM lineage and, for DNase-seq, also in heart. Boxes in (**F**,**G**), tissue-specific epigenetic marks at *KLHL40* seen in the *KLHL40*-expressing SkM lineage (purple) or in *KLHL40*-repressed heart and brain (blue). Dotted boxes in (**F**), tissue-specific H3K27ac at *HHATL* in SkM, heart, and brain, which selectively express this gene. Triangles, sites where the SkM lineage-specific MyoD transcription factor is bound.

*KLHL41* (formerly, *KBTBD10*), a gene whose recessive coding mutations can cause nemaline myopathy [10], had much higher expression in SkM (TPM, 3420) than did any other *KLHL* family gene and much lower expression in other tissues (Table 1 and Table S2, Figure 1A). It is also preferentially expressed in myoblasts relative to other cell cultures but displays much higher expression in myotubes (Table 1), suggesting that it is strongly upregulated upon myoblast fusion to form multinucleated myotubes. Consistent with its SkM lineage specificity, the SkM-specific transcription factor MyoD (Figure 1B, triangles over H3K27ac tracks) bound to sites 121 and 1294 bp upstream of the TSS) as well as to a site in intron 1, as determined by chromatin immunoprecipitation coupled with next-gen DNA sequencing in human myoblasts (MyoD ChIP-seq) [25]. These MyoD sites overlap regions of enhancer and promoter chromatin (Figure 1B, orange and red segments, respectively) and tissue-specific histone acetylation (Figure 1B, H3K27ac, purple box) in myoblasts, myotubes and SkM; the chromatin state profiles were generated by the Roadmap Project (see Methods). The most distal MyoD site overlaps a 0.2-kb hole in H3K27ac signal that probably corresponds to a nucleosome-depleted subregion. Such sites can be created by especially strong transcription factor binding to DNA. All three MyoD binding sites in myoblasts overlap tissue-specific peaks of open chromatin (Figure 1C, purple box). They typically correlate with such transcription factor binding sites within enhancer or promoter chromatin [26].

The strong histone acetylation (H3K27ac) signal seen in cells in the SkM lineage extends upstream of the *KLHL41* TSS to 5 kb downstream of it and indicates the presence of a super-enhancer (Figure 1B, blue dotted line). A super-enhancer is a long (>3 kb) cluster of enhancer or promoter chromatin that is associated with especially strong, differentiation-related expression of the gene regulated by this special type of enhancer [27,28]. Super-enhancers are especially densely occupied by transcription factors that have key differentiation- or tissue-specific functions and Mediator complex, which plays a central role in enhancer–promoter interactions via enhancer-bound transcription factors. Super-enhancers can be detected by unusually long regions of tissue-specific enrichment in H3K27ac (longer than 2 kb) than are found in traditional enhancers. They may also contain active promoter chromatin, which, like enhancer chromatin, exhibits enrichment in H3K27ac.

Overlapping most of the *KLHL41* super-enhancer was strong and SkM-specific DNA hypomethylation (Figure 1D, DNA meth, purple box) that was confirmed in two additional SkM samples from the Roadmap Project (data not shown). The unusual long region of DNA hypomethylation at mostly promoter-type chromatin (Figure 1B, SkM, red) in the super-enhancer may be responsible, in part, for the especially high level of expression of this gene. DNA-hypomethylated promoter chromatin extending far downstream from the TSS and associated with preferential expression in SkM was also seen in *KLHL33* and *KLHL31* (Supplementary Figures S1 and S2).

Heart had the second highest level of *KLHL41* expression among the 50 examined tissues (left ventricle TPM, 57, and right atrial appendage TPM, 48). Surprisingly, the heart and SkM DNaseI hypersensitivity profiles (Figure 1C) and their H3K4me3 profiles (data not shown) were very similar despite ~60-fold higher expression of *KLHL41* in these two tissues. However, heart lacked the strong extensive H3K27ac signal, the super-enhancer, and the tissue-specific DNA hypomethylation of SkM (Figure 1B,D). *KLHL41* displayed chromatin enriched in H3K36 trimethylation (H3K36me3) denoting actively transcribed chromatin in gene-upstream and downstream regions as well as in the gene body in most of the tissue and cell samples (Figure 1B, transcription-type chromatin (Txn-chrom), green segments). The H3K36me3 enrichment in Txn-chrom is characteristic of actively transcribed gene regions downstream of the 5′ end of the gene body. Surprisingly, this type of chromatin was seen even in samples with negligible RNA-seq levels for *KLHL41* (e.g., liver and leukocytes). Txn-chrom in tissues without appreciable levels of *KLHL41* RNA is probably due to low levels of read-through transcription from adjacent genes, in this case from *BBS5* (ENST00000513963/RP11-724O16.1), expressed at highest levels in testis or *FASTKD1*, whose 3′ ends are only 2–3 kb from the 5′ or 3′ ends of *KLHL41* (data not shown).

### 2.3. Intragenic and Intergenic Enhancer Chromatin at KLHL40 or Its Neighbor, HHATL May Upregulate Both Genes or One or the Other Gene Depending on the Tissue

*KLHL40* (formerly, *KBTBD*5), another gene that can contain recessive mutations causing nemaline myopathy [29], is the second most highly expressed *KLHL* gene in SkM (Figure 1E, TPM, 302). Like *KLHL41*, it is expressed specifically in SkM and in myotubes and, to a lesser extent, in myoblasts (Table 1). Only SkM and myotubes displayed much promoter chromatin near the TSS (Figure 1F). This promoter chromatin overlaps a CGI that had low levels of methylation in all tissues (Figure 1H) like most CGIs at promoters regardless of expression of the associated gene [14].

However, specifically in SkM, there was DNA hypomethylation in enhancer chromatin extending 0.3-kb upstream from the TSS as well as in a 1.5-kb enhancer chromatin region a little further upstream. Tissue-specific DNA demethylation extending from a constitutively unmethylated promoter CGI correlated with tissue-specific expression in nine other *KLHL* family genes, including *KLHL34* and *KBTBD12* (Supplemental Figures S3 and S4). In the promoter region of *KLHL40* at enhancer chromatin, there was a single site occupied by MyoD (Figure 1F, black triangle over H3K27ac tracks) [25]. This is one of several predicted MyoD sites in the~1.2 kb region upstream of the *KLHL40* TSS described by Bowlin et al. [30] and shown by them to be important for driving transcription of a reporter gene in mouse myoblast host cells. A second MyoD binding site described by Bowlin et al. (Figure 1F, blue triangle) was seen only in a ChIP-seq profiles of mouse myoblasts and myotubes [31].

Both heart and brain displayed enhancer chromatin over the *KLHL40* coding region (Figure 1F) even though they had negligible levels of RNA and little or no promoter chromatin for this gene (Supplemental Table S2). This heart and brain enhancer chromatin replaced a long segment of TSS-downstream promoter chromatin seen in SkM and is likely to be upregulating the *KLHL40*-upstream gene, *HHATL* in brain and heart (Figure 1E,F). Consistent with this proposal, based on chromatin conformation capture analysis of skin fibroblasts (Micro-C, [32]), *KLHL40* and *HHATL* are likely to be in the same transcriptionally associating domain (TAD) anchored by CTCF sites (Supplemental Figure S5) if the TAD structure in heart and brain cells is like that of fibroblasts. *HHATL* encodes a hedgehog acyltransferase that is involved in SkM maturation ([33]) and is present in the sarcoplasmic reticulum of SkM cells and cardiac myocytes [33]. It is expressed at high levels in heart, SkM, and brain. The only study of *HHATL* related to the nervous system reported changes in the levels of HHATL protein in myocardial tissue in a rat epilepsy model [34]. Interestingly, there is an antisense *HHATL* transcript (*HHATL*-*AS1*) whose expression is highest in SkM and brain (Figure 1E, RNA-seq bar graphs [19]). Specific enrichment in H3K27ac was seen in the *HHATL*/*HHATL*-*AS1* region in brain and SkM (Figure 1G, dotted boxes in H3K27ac tracks). Therefore, *HHATL*-*AS1*, like many long intergenic non-coding RNA genes 5′ to a coupled protein-encoding gene, may upregulate expression of *HHATL* specifically in SkM and brain but not in heart.

A third *KLHL* family gene associated with autosomal nemaline myopathy is linked to dominant mutations in *KBTBD13* [35]. Unlike the previously described genes, *KBTBD13* is very small (3.1 kb) with no introns (Supplemental Figure S6) and is expressed only at a low levels in SkM (TPM, 1.4) although the expression ratio in SkM to the median of non-SkM tissues is 25 (Table 1). *RASL12*, which encodes a small GTPase superfamily protein, is expressed antisense to *KBTBD13.* It has an isoform (ENST00000434605) with a TSS that is only 0.1 kb upstream of the *KBTBD13* TSS. This isoform and *KBTBD13* have partly overlapping expression profiles, which is not surprising given their shared core promoter region. Although *KBTBD13* is not expressed in myoblasts (Table 1) or myotubes, there were several occupied MyoD binding sites downstream of the gene in these cells (Supplemental Figure S6, above H3K27ac tracks), which may be used for activation of this gene in later stages of myogenesis.

### 2.4. Skeletal Muscle-Specific Expression, Promoter Chromatin, and Enhancer Chromatin in KLHL30 and in KLHL38, a Paralog That Contains a Retrogene from KLHL30

**Figure 2:**
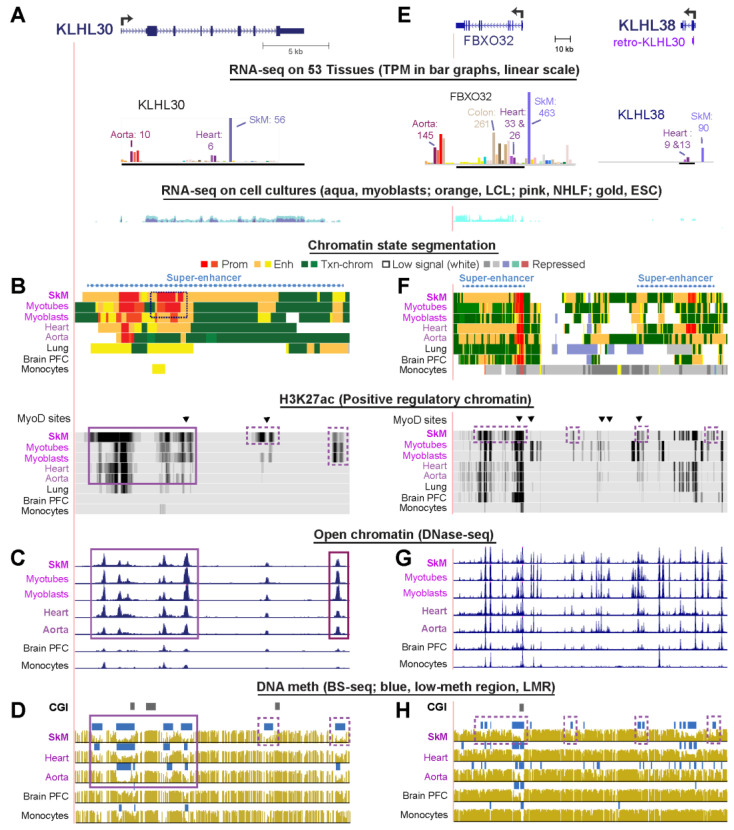
*KLHL30* and *KLHL38*, little studied *KLHL* genes, have super-enhancer chromatin and DNA hypomethylation that can drive their high, muscle-specific expression. (**A**–**D**) *KLHL30* (chr2:239,043,816-239,065,093) and (**E**–**H**) *KLHL38* and its downstream neighbor, *FBXO32* (chr8:124,508,193-124,686,846). Tracks from UCSC Genome Browser are as described in Figure 1. The immediate upstream neighbor of *KLHL38*, *FBXO32*/Atrogen-1, a gene encoding an E3-ubiquitin ligase, is implicated in muscle atrophy [37]. Boxed regions are tissue-specific epigenetic marks described in the text; triangles, sites where MyoD is bound.

*KLHL30*, a gene that has not been the focus of any published articles, is much more highly expressed in SkM than in any other tissue and has higher expression in myoblasts and myotubes than in non-muscle cell cultures (Figure 2A; Table 1). Like *KLHL41* and *KLHL40,* its high expression in SkM is associated with a SkM-specific super-enhancer (Figure 2B, dotted line above chromatin state tracks). This super-enhancer contained strong promoter chromatin in several regions in the first half of the gene body. This intragenic promoter activity may drive unusually high levels of expression of noncoding RNAs (ncRNAs) associated with active enhancers [36]. Heart and aorta, which lacked the super-enhancer and intragenic promoter chromatin, had considerable expression of this gene, although approximately 5–10 times less than that of SkM. SkM, heart, and aorta had similar open chromatin profiles (Figure 2C). Heart and aorta also lacked a H3K27ac-rich chromatin region within *KLHL30* and another downstream of the end of the gene that overlapped DNA hypomethylation in SkM (Figure 2B,D, dotted boxes). There were two MyoD binding sites within the super-enhancer in myoblasts overlapping SkM-hypomethylated regions (Figure 2B, triangles over H3K27ac tracks).

There is an intriguing relationship between *KLHL30* and *KLHL38*, another little studied gene. One report suggests the encoded protein may reverse muscle atrophy [38]. Most of the open reading frame (ORF) of *KLHL38* is in exon 2 and is largely derived from a retrotransposed copy (retrogene) of coding sequences in exon 2 of *KLHL30* (Figure 2E). Both genes have a strong preference for expression in SkM (Table 1). The retrogene from *KLHL30* located in *KLHL38* encodes the BTB/POZ and Back domains and two of the Kelch repeats [6] as determined by protein BLAST (BLASTp). There are eight other *KLHL* family genes containing a retrogene exon from exonic DNA of another *KLHL* gene (Supplemental Table S3). The *KLHL30* retrogene in exon 2 of *KLHL38* is an expressed shuffle retrogene, i.e., a retrogene that is expressed in a pre-existing host gene and contributes part of its ORF to the host gene’s ORF [24,39]. Five other *KLHL* family genes contained expressed shuffle retrogenes derived from other *KLHL* family members (Supplementary Table S3). Three additional *KLHL* family genes contained retrogenes derived from another *KLHL* family member but did not meet the definition of an expressed shuffle retrogene. The retrogene in *KLHL38* had an especially high percentage sequence identity to its parent gene, namely, 80%, suggesting that it is the result of a rather recent retrotransposition during evolution. In comparison, among the 740 host genes in the human genome containing expressed shuffle retrogenes, the median percentage of bases matching the parent gene was only 31% (Supplementary Table S4). These findings are consistent with KLHL38 and KLHL30 coming from the same clade (Clade 4, Supplemental Figure S7; see [7] for a similar analysis that did not identify clades).

Like *KLHL30*, *KLHL38* was embedded in a super-enhancer in SkM (Figure 2F). *KLHL38* also displayed a super-enhancer in heart even though the steady-state *KLHL38* RNA levels in heart were only modest, unlike those for SkM. However, the heart super-enhancer extended only 2 kb upstream of the TSS while the SkM enhancer began 13 kb upstream. Importantly, heart had fewer subregions of DNA hypomethylation and less H3K27ac enrichment in the intergenic region between *KLHL38* and its downstream neighbor *FBXO32* (Figure 2F,H), dotted boxes). In the 1-Mb neighborhood of *KLHL38*, *FBXO32* (Atrogin-1) was the only other gene exhibiting strong preferential expression in SkM, and, like KLHL38 is embedded in a super-enhancer. *FBXO32* is critically involved in the ubiquitin-driven degradation of proteins in SkM during sarcopenia ([40]. It is expressed most highly in SkM and at an even higher level than *KLHL38*, but at much lower levels in heart (Figure 2E). In the *KLHL38*/*FBXO32* intergenic region, MyoD binding was seen at four sites in human myoblasts (Figure 2E, black triangles over H3K27ac tracks), which express *FBXO32* but have only barely detectable expression of *KLHL38* (Figure 2E and Table 1). This suggests that the MyoD binding sites and enhancer chromatin in the intergenic region between *FBXO32* and *KLHL38* are upregulating just *FBXO32* in these SkM progenitor cells. Similarly, aorta strongly expresses *FBXO32* but weakly expresses *KLHL38* and has extensive enhancer chromatin and tissue-specific foci of DNA hypomethylation in the intergenic region. Moreover, aorta also exhibited DNA hypomethylation in the gene body of *KLHL38* and the *KLHL38*-upstream region, which probably also upregulates only *FBXO32*. In SkM, there might be long-distance interactions between promoters, super-enhancers, and hypomethylated enhancer segments between the two super-enhancers that are responsible for the very high levels of expression of both genes specifically in skeletal muscle. Findings from chromosome looping analysis are consistent with this hypothesis because the *KLHL38*/*FBXO32* neighborhood is in a TAD of ~225 kb ending at CTCF sites as determined in skin fibroblasts (Micro-C [32], Supplemental Figure S8).

### 2.5. Only Minor Skeletal Muscle-Associated Epigenetic Differences Occur in KEAP1 Consistent with Its Highest Expression in SkM But Otherwise Broad Tissue Expression Profile

**Figure 3:**
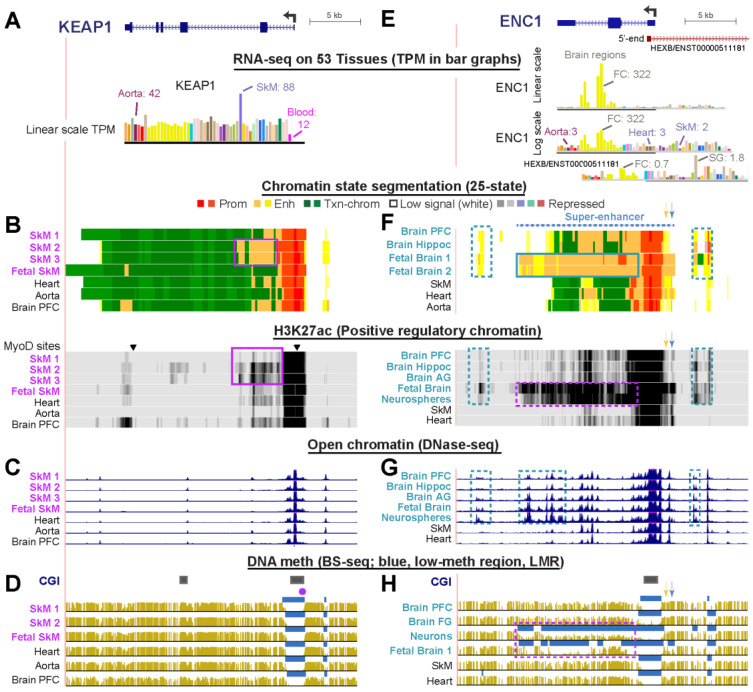
Epigenetic profiles for *KEAP1*/*KLHL19* and *ENC1*/*KLHL37*, which encode critical proteins that bind to NRF2 and regulate adaptation of cells to oxidative stress. (**A**–**D**) *KEAP1* (chr19:10,591,087-10,619,523) and (**B**–**H**) *ENC1* (chr5:73,908,815-73,950,517). Tracks are shown as in Figure 1. SkM 1 refers to psoas muscle while SkM 2 and SkM 3 are SkM from upper leg [4,15]. Boxed regions are tissue-specific epigenetic marks; orange and blue arrows are previously reported human phenotype-linked hypomethylated CpG sites described in the text. FC, frontal cortex; SG, salivary gland; PFC, prefrontal cortex; Hippoc, hippocampus; AG, angular gyrus; FG, frontal gyrus.

*KEAP1* (Kelch Like ECH Associated Protein 1, *KLHL19*) plays a major role in regulation of cellular oxidative stress in SkM and other tissues and contributes to cancer and other diseases [6] (Supplemental Table S5). KEAP1 protein participates in the oxidative stress response by targeting the transcription factor NFE2L2 (NRF2) for degradation [41]. Given the protective role of KEAP1, it is not surprising that the gene is expressed with a broad tissue distribution (Figure 3A). Nonetheless, SkM has a higher level of *KEAP1* RNA compared with other normal tissues and an expression ratio of 2.3 for SkM TPM relative to the median TPM of 51 other tissues (Figure 3A, Table 1, and Supplemental Table S2). Chromatin segmentation, open chromatin, and DNA methylation profiles (Figure 3B–D) in and around this gene were similar in all tissues except that two of the three SkM samples (SkM 2 and SkM 3) for which histone modification profiles were available had more enhancer chromatin and H3K27ac signal in the promoter-downstream region than seen in the other samples (Figure 3B, purple boxes). The main Roadmap sample (SkM 1) is from psoas (trunk) muscle, but the source of the SkM of these two samples is from unspecified muscle types in the upper leg. The 803 SkM samples used to determine the median RNA levels for the GTEx database were from gastrocnemius (calf) muscle samples. Differences in the muscle subtypes examined could account for differences in chromatin state profiles of these three muscle samples. The exact source of SkM likely affects its *KEAP1* expression as it does for some other genes [42]. Our analysis suggests that for expression of *KEAP1*, SkM 2 and 3 are more like gastrocnemius muscle than like psoas.

### 2.6. The Neurogenesis-Associated ENC1 Gene Exhibited Much More Enhancer Chromatin and DNA Hypomethylation in Fetal than in Adult Brain

*ENC1* (Ectodermal-Neural Cortex 1; *KLHL*37, Figure 3E) is the most highly expressed *KLHL* family gene in the frontal cortex and hippocampus of brain (TPM, 322 and 87, respectively) (Table 2 and Supplemental Table S2). Like *KEAP1*, *ENC1* helps control the oxidative stress response as a post-translational regulator of the transcription factor Nrf2 [43] and is expressed in all tissues (Figure 3E). However, it is much more highly expressed in the frontal cortex of brain than in non-neural tissues. *ENC1* also plays an important, but poorly understood, role in prenatal neural development [44]. The gene’s most prominent postnatal brain-specific epigenetic marks were the weak or strong enhancer chromatin upstream, H3K27ac signal, and downstream intergenic open chromatin (Figure 3F,G, blue dotted boxes). The higher level of expression of *ENC1* during prenatal brain formation than in adults [44] is matched by the strong H3K27ac signal and fetal brain-associated DNA hypomethylation in the gene body (Figure 3F,H, purple dotted boxes) as well as a super-enhancer spanning the gene (Figure 3F, dotted line). The extensive H3K27ac enrichment and DNA hypomethylation were also seen in NeuN/RBFOX3-antibody isolated postnatal neurons [45] or neurospheres (suspended in vitro clusters of neural progenitor cells (Figure 3F,H, purple dotted boxes). Non-neuronal cells from postnatal brain [45] lacked the prominent and specific DNA hypomethylation of neurons and fetal brain (Figure 3H and data not shown). Importantly, *ENC1* was the only one of the six *KLHL* family genes with brain-preferential expression that displayed a consistent difference in DNA methylation profiles with age. A 35-day postnatal sample from frontal cortex displayed the same DNA hypomethylation seen in a 17-week fetal cerebral cortex but this hypomethylation was missing in samples of frontal cortex from donors 2, 12, 16, 53, 55, or 64 years of age (Figure 3H and data not shown). Two population studies of genetic and epigenetic correlates of neurological function gave evidence for differential methylation of several CpGs 1 to 2 kb upstream of the *ENC1* TSS being associated with cognitive decline or schizophrenia (Figure 3F, orange and blue arrows, positions of CpGs in studies by White et al. [46] and van den Oord et al. [47], respectively). Interestingly, these CpGs are in a region that shows the most enhancer chromatin signal and the lowest DNA methylation in fetal brain (Figure 3F,H, respectively).

A caveat in studies of DNA methylation in brain is that bisulfite-seq, like most DNA methylation analysis techniques, cannot distinguish between 5-methylcytosine (5mC) and 5-hydroxymethylcytosine (5hmC). Brain tissue is particularly rich in genomic 5hmC [45] although other tissues, like SkM, are also enriched in 5hmC at tissue-specific loci [48]. Nonetheless, the main attributes of DNA methylation patterns that we observed are tissue-specific differences in the presence of low-methylated regions (LMRs), which are independent of whether the surrounding DNA is rich in 5mC or 5hmC. In addition, complications like allele-specific differences in DNA methylation [49] will not affect detection of regional DNA hypomethylation.

*ENC1* displays moderately high expression and much enhancer chromatin in several primary cultures or progenitor cells unrelated to brain (Table S2). This is in accord with reported additional developmental roles for *ENC1* in non-brain tissues [50]. There is a long isoform of *HEXB* (*ENST00000511181*), which is antisense to *ENC1* and whose TSS overlaps the first exon of *ENC1.* However, this RNA is expressed at only very low levels in brain and has a tissue profile with only slight similarity to that of ENC1 (Figure 3E).

### 2.7. The Strong Brain-Specificity of KLHL32 Is Mirrored by Clusters of Intragenic Enhancer Chromatin Seen Only in Brain

**Figure 4:**
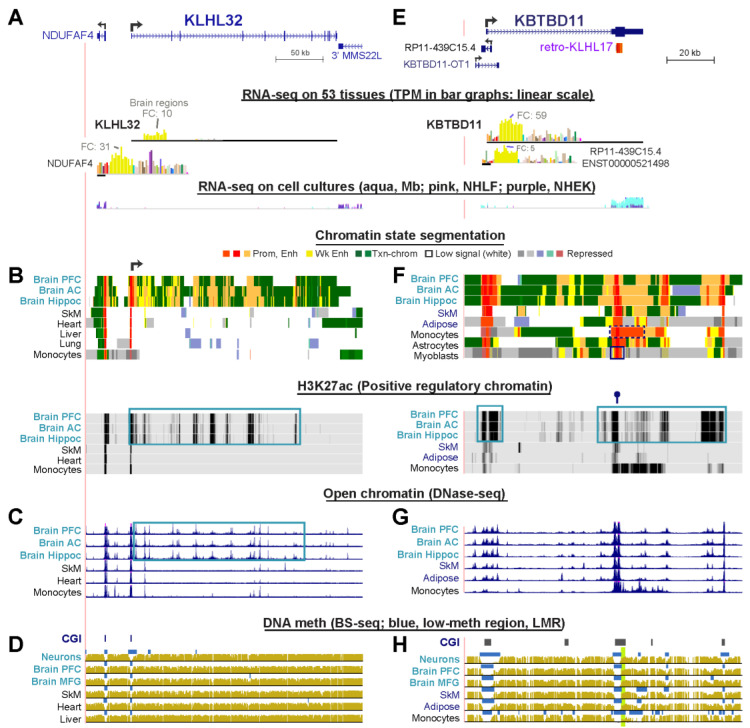
Multiple segments of enhancer chromatin without brain-specific DNA hypomethylation are associated with highly preferential expression in brain of *KLHL32* and *KBTBD11*. (**A**–**D**) *KLHL32* (chr6:97,324,594-97,615,081) and (**B**–**H**) *KBTBD11* (chr8:1,917,290-1,976,132). Tracks are as in Figure 1. The GTEx RNA-seq bar graphs are in linear scale but the scale for *KBTBD11* is different from that of its 5′ overlapping antisense transcript RP11-439C15.4, as indicated by the TPM values for frontal cortex. The green highlighting in the DNA methylation tracks for *KBTBD11* refers to a region with leukemia-subtype-related DNA hypomethylation as previously reported [52] and explained in Section 3. Boxed regions are tissue-specific epigenetic marks described in the text; PFC, prefrontal cortex; FC, frontal cortex; AC, anterior cortex; MFG, midfrontal gyrus; Hippoc, hippocampus.

The steady-state RNA levels from *KLHL32*, a gene which has received very little attention, ranged from TPM 7.6 to 25.2 in different brain tissues and spinal cord in contrast to 0 to 2.6 for 39 other tissues (Figure 4A, Supplementary Table S2) Expression was negligible in various cell cultures unrelated to brain. In accord with its nervous system-related transcription, *KLHL32* exhibited brain-specific enhancer chromatin segments overlapping brain-specific DNase-seq peaks and H3K27ac peaks throughout its long gene body (Figure 4B,C, blue boxes). In addition, all brain samples had a broadened promoter chromatin region at the TSS (Figure 4B, broken arrow). Two samples of neurons isolated from adult brain displayed a large broadening of the DNA hypomethylation at the CGI promoter (Figure 4D and data not shown). This hypomethylation was not seen in nonneuronal cells [45] from the brains of the same individuals nor in fetal brain. Fetal brain also lacked most of the enhancer chromatin seen in postnatal brain. The main TSS of *KLHL32* [19] is 25 kb from the TSS of *NDUFAF4*, which encodes a mitochondrial NADH:Ubiquinone oxidoreductase complex assembly factor. *NDUFAF4* is broadly expressed but its RNA levels are highest in frontal cortex (TPM, 31 vs. median TPM of 2.5 for non-brain tissues). *NDUFAF4* displayed brain-specific enhancer chromatin immediately upstream of its promoter (Figure 4B). Therefore, the brain-specificity of *KLHL32* has its counterpart in the brain-preferential expression and epigenetics of *NDUFAF4*, although it is unclear if they influence each other, because in skin fibroblasts, they are in different TADs (data not shown).

### 2.8. KBTBD11, a Brain-Specific Gene, Has a Retrogene Overlaying a 3′ Promoter for a Novel Noncoding RNA Gene

*KBTBD11* has been little studied in brain but is implicated in adipocyte differentiation [51]. It has moderately low expression in normal subcutaneous or visceral adipose tissue (TPM, 4–6), as in most other non-brain tissues (Figure 4E). We found that this gene is preferentially expressed in all brain tissues (TPM, 23–59; Supplemental Table S2) and its expression correlated with brain-specific enhancer chromatin and H3K27ac enrichment (Figure 4F, orange segments and blue boxes) with only modest brain-specificity for open chromatin profiles (Figure 4G). Fetal brain, for which expression data are not available, had much less enhancer chromatin (data not shown) than postnatal brain. Even though there was negligible expression of *KBTBD11* mRNA in SkM, there was moderate and selective expression in myoblasts (Figure 4E, RNA-seq profiles for cell cultures and Supplemental Table S2) and osteoblasts (UCSC Genome Browser, data not shown). However, much of this transcription in myoblasts and osteoblasts uses an intragenic TSS in the last exon of *KBTBD11*, in the sense direction. This was indicated by analysis of 5′ cap ends of RNA (5′ Cap Analysis Gene Expression, CAGE, not shown [24]) and confirmed by RNA-seq and active promoter chromatin at the end of the gene (Figure 4F, black box in last chromatin state track). In addition to myoblasts and osteoblasts, monocytes displayed especially strong expression of just this 3′ exonic ncRNA, which is consistent with monocytes’ unusually long region of promoter chromatin at the 3′ end of the gene (Figure 4F, dotted black box in chromatin state tracks). The 3′ region of *KBTBD11* overlaps a CGI with constitutive lack of methylation but this unmethylated region is broader specifically in monocytes (Figure 4H, last track). Monocytes displayed clusters of TSSs for the plus strand in this region but not at the 5′ end of *KBTBD11* (CAGE data, not shown). The ORF of *KBTBD11* is fully contained in the final exon of this gene. Curiously, most of this ORF overlaps a *KLHL*-derived retrogene, *retro-KLHL17* (Figure 4E, top). However, the ORF of this retrogene deviates so much from that of its parent gene that it is not classified as an expressed shuffle retrogene (Supplemental Table S3). The above-mentioned 3′ promoter chromatin, which was seen in many types of tissues and cell cultures, overlaps the retrogene. A central CTCF binding site was observed in the retrogene in some samples regardless of their *KBTBD11* expression status (Figure 4F, lollipop over H3K27ac tracks [25]). This undocumented monocyte/myoblast/osteoblast ncRNA transcribed from the 3′ end of *KBTBD11* may encode a miRNA sponge [3]), given that its cell type-specific transcription is unrelated to the tissue/cell specificity of *KBTBD11* expression. There is, in addition, an antisense ncRNA gene *RP-11-439C15.4* overlapping the beginning of the first intron of *KBTBD11* (Figure 4E, top). Its tissue-specific expression profile is very similar to that of *KBTBD11*, although it is expressed at lower levels and so it probably helps regulate the transcription of its overlapping protein-encoding gene. We could find no evidence of transcription of another ncRNA gene, *KBTBD11-OT1* (Figure 4E), in any of the studied tissues or cell lines.

### 2.9. Overview of Epigenetic Features Associated with the Studied KLHF Family Genes

As shown in Table 3, enhancer chromatin, expanded promoter chromatin at the 5′ end, and/or DNA hypomethylation specific for SkM or brain correlated with the preferential expression of 17 *KLHL* family genes in SkM or brain. SkM or brain DNaseI-hypersensitive sites usually, but not always, were seen in the tissue-specific enhancer or promoter chromatin and were frequently at SkM- or brain-specific peaks of H3K27ac signal (Figure 1, Figure 2, Figure 3 and Figure 4). Posttranscriptional regulation was not examined in this study but is, of course, widespread. microRNAs (miRNAs) that fine-tune mRNA levels of *KLHL* family genes were previously described for six of the genes examined in our study (Supplemental Table S5). However, despite the importance of miRNAs regulating mRNA stability and translation, our results clearly establish that there are important epigenetic contributions to regulation of tissue-specific transcription of these *KLHL* family genes.

## 3. Discussion

The preferential expression of 17 *KLHL* family genes in SkM or brain (Table 1 and Table 2 and Supplemental Tables S1 and S2) is matched by the SkM- or brain-specific intragenic enhancer chromatin seen for almost all of these genes (Table 3). Their epigenetic specificity is consistent with their known tissue-specific roles in targeting certain proteins for degradation [6]. One exception is *KLHL35*, which although preferentially expressed in brain, exhibited little or no observable intragenic enhancer chromatin (Supplemental Table S2). Most of the examined *KLHL* family genes also displayed SkM- or brain-specific enhancer chromatin upstream or downstream of the gene. The intergenic and intragenic enhancer chromatin usually contained foci of DNA hypomethylation but occasionally a large region of DNA hypomethylation overlapping extended enhancer chromatin was seen throughout much of the gene body, especially for *KLHL41* in SkM and *ENC1* in fetal brain (Figure 1D and Figure 3H). These exceptions to generalizations about higher expression being associated with higher intragenic DNA methylation [4] and enhancer DNA hypomethylation usually being limited to foci [15] illustrate the importance of examining epigenetic profiles of individual genes to gain insight into their transcription regulation. As has been found for some other gene families [16], in the *KLHL* gene family, tissue-specific enhancer chromatin was more common than tissue-specific promoter chromatin. About half of these genes displayed SkM- or brain-associated demethylation extending upstream and/or downstream from a constitutively unmethylated CGI at the promoter region.

Promoter–enhancer interactions that boost transcription initiation are mediated by looping of the enhancer to its target promoter, which may facilitate their sharing of bound transcription factors and cis-acting short-lived ncRNAs [36]. Such sharing is facilitated by a local lack of DNA methylation but is interrupted by insulator-type loop boundaries. Therefore, the enhancer–promoter pairs should be in the same topologically associating domain (TAD) for effective enhancer action [53]. We found evidence consistent with long-distance enhancer-promoter interactions that involve an enhancer within one gene and a promoter of another gene, namely, *KLHL40* with *HHATL* and *KLHL38* with *FBXO32* (Figure 1 and Figure 2; Supplemental Figures S5 and S8). The 3′ end of *KLHL40* is only 0.1 kb from the 3′ end of *HHATL* and both have high and preferential expression in SkM but *HHATL* is also expressed in heart. Hedgehog acyltransferase like (HHATL) is needed for normal postnatal skeletal muscle maturation [33] while mutation of *KLHL40* is linked to Nemaline Myopathy 8 and Severe Congenital Nemaline Myopathy reflecting the role of KLHL40 in regulating skeletal muscle development [29]. The TSS-downstream promoter chromatin of *KLHL40* in SkM appears as enhancer chromatin associated with *HHATL*-upregulation in heart (Figure 1F). This dual function of a region as a promoter or enhancer for expression of the *KLHL* family genes depending on the tissue type is consistent with genome-wide studies [54]. In addition, we propose that, in SkM, there are cooperative interactions between enhancers overlapping *KLHL40* and *HHATL* to potentiate expression of both genes via a large super-enhancer, just as cooperative enhancer-enhancer interactions have been inferred elsewhere in the human genome [55].

*KLHL38* and *FBXO32* (Muscle atrophy F-box Protein) (Figure 2E) is the other gene pair that we predict have shared enhancer-enhancer interactions. These interactions could span their shared 103-kb intergenic region that overlaps multiple MyoD sites and SkM-specific DNA hypomethylated regions (Figure 2E–H). Both proteins encoded by these genes are involved in ubiquitin-mediated protein degradation. FBXO32 is well known for its major role in SkM atrophy [56]. In a rat castration-reversal model of muscle atrophy, *Klhl38* was upregulated while its neighbor *Fbxo32* was downregulated [38]. However, in humans, both genes have their highest expression in normal SkM, and their intragenic and intergenic enhancer chromatin could positively interact with each other (Figure 2 and Supplemental Figure S8). Consistent with cooperative regulation of these genes, both *klhl38* (*klhl38b*) and its neighbor *fbxo32* in zebrafish are upregulated by glucocorticoids and may be involved in glucocorticoid-mediated muscle atrophy [56].

The most dramatic enhancer cooperation is seen in genes with super-enhancers. Super-enhancers are large structures consisting of clusters of neighboring enhancers (or enhancers and promoters) that usually are associated with strongly upregulating expression of differentiation-related genes [27], including SkM-associated genes [15,16]. Seven of the 17 SkM- or brain-associated *KLHL* family genes exhibited expression-linked super-enhancers (Table 3). Four of these genes, *KLHL40*, *KLHL41*, *KLHL31*, and *ENC1*, are known to be important for SkM or brain development [29,44,57]. The other three genes, *KLHL21*, *KLHL30*, and *KLHL38*, have been the subject of only a few studies and their role in early development is not known. The presence of super-enhancers in SkM and the high expression in myoblasts and myotubes or, for *KLHL38*, just in myotubes (Table 1) as well as in postnatal SkM suggests that these three genes have as yet unrecognized roles in muscle development and in SkM homeostasis. The above-postulated *KLHL38*-*FBXO32* enhancer interactions in SkM would be between two neighboring super-enhancers. Super-enhancers may help to compartmentalize transcription regulatory factors by a type of localized phase separation [58]. Focal cell/tissue-specific DNA hypomethylation is considered to be an important hallmark of super-enhancers (and traditional enhancers [59]) by helping to facilitate the concentration of transcription factors at some of their most influential subregions. We found such SkM-specific hypomethylated foci in SkM super-enhancers, except for *KLHL41*, which has a super-enhancer that consisted mostly of unusually extensive promoter chromatin that was hypomethylated over most of its length. Both epigenetic features correlate with *KLHL41′*s extremely high expression.

Surprisingly, a promoter chromatin region overlapped the last exon of *KBTBD11* (Figure 4F). This 3′ promoter chromatin likely evolved from a retrotransposed copy (retrogene) of *KLHL17*. In addition, *KLHL31* similarly has a *KLHL* retrogene (*retro-KLHL36*) at its 3′ end that is embedded in an open reading frame (Supplemental Figure S2). The *KLHL31* retrogene overlaps exonic promoter chromatin in *KLHL31*-expressing and non-expressing tissues. Importantly, both the *KLHL31* and *KBTBD11* intragenic retrogenes are in CGIs that are constitutively unmethylated, and so both retrotransposed sequences are predisposed to forming intragenic promoters [60], and, indeed, both had evidence of transcription initiation in some normal cell cultures based on CAGE profiling. Of the nine SkM- or brain-associated *KLHL* genes that contained a *KLHL* retrogene in their exonic DNA (Supplemental Table S3), only one other gene, *KLHL21*, had a constitutively unmethylated CGI overlapping the retrogene, but this retrogene was in the first exon rather than at the 3′ end of the host gene. Therefore, it is not surprising that the retrogene in *KLHL21* overlapped promoter chromatin in all tissues (Supplemental Figure S10). The three other *KLHL* family genes (*KLHL17*, *KLHL25*, and *KLHL2*6) that contained intragenic CGI-overlapping *KLHL* retrogenes had their retrogenes embedded in transcription-type chromatin (enriched in H3K36me3) that is highly and constitutively methylated in the CGI. This constitutive DNA methylation is consistent with evidence for DNA methylation repressing the latent promoter activity of intragenic CGIs [61], just as hypermethylation of constitutively unmethylated and active promoter regions overlapping CGIs almost invariably leads to repression [62].

The importance of having normal levels of expression of *KLHL* family genes is supported by small interfering RNA (siRNA) knockdown or overexpression studies in which decreases or increases in protein levels of the *KLHL* family genes impacted normal cellular function (e.g., for *KLHL40*, *KLHL41*, *KEAP1*, *ENC1,* and *KBTBD11* [30,63,64,65,66]). Chromatin and DNA epigenetics are frequently involved in setting or maintaining gene expression levels [4,16,62]. In addition, fine-tuning of RNA abundance by downregulation at the post-transcriptional level is often mediated by miRNAs. Such post-transcriptional regulation is attested to by reports of miRNA regulation of *KEAP1*, *ENC1*, *KLHL2*, *KLHL31*, *KBTBD11*, and *KBTBD12* expression (Supplemental Table S5).

Most epigenetic studies of *KLHL* family genes focused on cancer-associated promoter hypermethylation, especially, hypermethylation of the *KEAP1* CpG island promoter during carcinogenesis or acquisition of drug resistance [6]. Under homeostatic conditions, KEAP1 is a major regulator of antioxidant and metabolic genes by continuously ubiquitinating and targeting for degradation the oxidative-stress responsive transcription factor NFE2L2/NRF2 [41]. Increases in NRF2 levels can result from KEAP1 inactivation by the reaction of electrophiles or oxidants with cysteine residues in KEAP1 or by disruption of the KEAP1:NRF2 complex [21]. It can also occur by extensive *KEAP1* promoter hypermethylation, as has been described in diverse cancers [67,68]. The resulting increase in NRF2 protein levels can affect tumor initiation, tumor progression, and drug resistance. It has been reported that there is cataract-associated DNA hypomethylation in lens epithelial cells at the TSS-upstream portion of the 5′ *KEAP1* CGI [67], which is surprising because the analyzed region described as highly methylated in normal lens had low levels of methylation in more than 20 examined human tissues with bisulfite-seq profiles at the UCSC Genome Browser (Figure 3D, purple circle; [24]). However, a methylome profile for lens tissue was not available, and *KEAP1* methylation might be exceptional in this subregion. What is clear is that cellular levels of KEAP1 must be tightly regulated and the first step in such regulation is by epigenetic control of the gene’s transcription.

Examination of *KBTBD11* epigenetics gives another example of how using publicly available databases can elucidate epigenetic changes in normal and diseased tissues. In one of the few studies of non-promoter epigenetics of *KLHL* family genes, Kachroo et al. [52] found that a 0.8-kb region in the last exon of *KBTBD11* was significantly hypomethylated in lymphoblasts from untreated pediatric patients with a poor-prognosis subtype of B-cell leukemia relative to those with a good-prognosis leukemia subtype. DNA hypomethylation at this 0.8 kb region in the poor prognosis-subtype correlated with increased expression of *KBTBD11*. We found that this region in the ORF of the last exon of *KBTBD11* was hypomethylated in normal monocytes relative to other studied normal samples (Figure 4H, green highlighting). Moreover, the monocyte hypomethylation is associated with production of a novel ncRNA from the 3′UTR with a different tissue-specificity from that of *KBTBD11*. Therefore, the epigenetics, transcription, and function of this previously unknown ncRNA gene encoded at the 3′ retrogene promoter of *KBTBD11* should be examined in future studies of normal and cancer samples. Our results indicate the need for follow-up studies of the effects of experimental manipulation of some of the most tissue-specific of the enhancer chromatin regions that we characterized, especially those that are correlated with disease.

## 4. Methods

### 4.1. RNA-Seq for Tissues and Cells

TPM values for RNA levels for tissues were from the GTEx RNA-seq database (Supplemental Table S2) [19]. The median TPM values are from analysis of hundreds of samples for each of 52 tissue types. In GTEx graphs, heart indicates two tissue types: left ventricle and right atrial appendage. We included only GTEx data for tissues and not the two cell lines listed in the database. For genes with more than one isoform, except where otherwise noted, only the main transcribed isoform is shown in the figures, as deduced from GTEx isoform expression profiles, the position of TSS-overlapping promoter chromatin, or the TSS deduced by 5′ Cap Analysis of Gene Expression (CAGE; RIKEN Omics Science Center [24]). RNA-seq data (FPKM) for human cell cultures are in Supplemental Table S2. The human cell cultures are normal myoblasts, GM12878 (a lymphoblastoid cell line), embryonic stem cells (ESC), human umbilical vein endothelial cells (HUVEC), normal human epidermal keratinocytes (NHEK), and normal human lung fibroblasts (NHLF) [24]. The quantitation of cell culture RNA-seq data employed the Cufflinks tool as previously described [69,70]. For comparisons of RNA levels in myoblasts and myotubes, we used our previously generated data [16].

### 4.2. Databases and Analyses Used for Epigenetics Studies

The 18-state Roadmap Epigenomics chromatin state segmentation analysis (chromHMM, AuxilliaryHMM) [4]) was used for determination of chromatin states (promoter, enhancer, repressed, etc.) except for *KEAP1* and *ENC1* in which the 25-state analysis was used because fetal brain is included only in that dataset. The color code for chromatin state segmentation in the figures was slightly simplified from the original as shown in the color keys in the figures. The chromatin state profiles had been derived as part of the Roadmap Project from global maps of ChiP-seq signal from H3K27ac, H3K4me1, H3K4me3, and two predominantly repressive H3 modifications, H3K27me3 and H3K9me3 using a multivariate Hidden Markov Model and a model learning procedure [4]. Strong enhancer or promoter chromatin (Prom, Enh, red and orange segments, respectively; Figure 1B and F) displays a moderate or strong signal for both H3K27ac and H3K4me1 or for both H3K27ac and H3K4me3, respectively [4]. Weak enhancer chromatin (wk enh, yellow segments) has only a low H3K27ac signal but a considerable H3K4me1 signal. Chromatin enriched in H3K36me3 is seen in the gene body of most actively transcribed genes but not in the immediate TSS-downstream region (Txn-chrom, green segments). The chromatin segments denoted as repressed in the figures include bivalent (paused) enhancer chromatin (light blue/purple segments) or bivalent promoter chromatin (brown segments), which contain H3K27me3 (associated with repression) and H3K4me1 or 3 (associated with promoter or enhancer chromatin when H3K27ac is also present).

Bisulfite-seq profiles of genome-wide CpG methylation and the DNaseI-hypersensitivity profiles were also from the RoadMap Project [4]. For bisulfite-seq samples of SkM, heart, and brain, several biological replicates were available as follows: SkM, psoas from a 3 year-old male, 30 year-old female, or 34 year-old male; left or right ventricle from the former two donors or right atrium from the latter donor; brain prefrontal or midfrontal cortex from seven female or male donors ranging from fetal to 55 years old. Except for a few genes preferentially transcribed in brain noted in Section 2, similar results were obtained from these biological replicates. With the exception of brain, and sometimes SkM, generally the same samples that had been used for bisulfite-seq were used for chromatin state segmentation (and the associated H3K27ac signal) and DNase-seq. SkM 1 refers to psoas (combined 3 year-old and 34 year-old) and SkM 2 and 3 to leg muscle (unspecified) from a 72 year-old female and a 54 year-old male, respectively. Fetal muscle was from a 15-week post gestational male or female. In the epigenetic tracks in the figures, SkM and heart with no further designation refer to mixtures of psoas muscle or left ventricle from the 3 year-old and 34 year-old. Additional descriptions of other samples used for these epigenetic analyses were given previously [4,48]. Super-enhancers were assessed by dbSUPER [71] and confirmed by looking at the H3K27ac track in the UCSC Genome Browser [24]. MYOD binding sites in myoblasts were determined from the Unibind data track in the UCSC Genome Browser [24,25]. Low methylated regions (LMRs) shown in the figures refer to regions with significantly lower DNA methylation than in the rest of the genome for the same tissue sample as determined by Song et al. [72]. Transcription associating domains (TADs) were obtained using the Micro-C tracks for foreskin fibroblasts at the UCSC Genome Browser [73]. A high score between two regions suggests that they are probably in proximity in 3D space within the nucleus of a cell as indicated by an arc with more intense color in the heatmap [73].

### 4.3. Alignments and Phylogenetic Analysis

Alignments of the main isoform for each of the *KLHL* family genes was done using the phylogenetic tree view in COBALT (COnstraint-Based multiple ALignment Tool, with the default parameters: fast minimum evolution, Max seq difference 0.85, distance, Grishin (for protein) [74] https://www.ncbi.nlm.nih.gov/tools/cobalt/cobalt.cgi?LINK_LOC=BlastHomeLink.

## 5. Conclusions

Analysis of *KLHL* family genes preferentially expressed in SkM or brain showed that differences in the amount of enhancer chromatin are more likely to be indicative of tissue-specific differences in mRNA levels than are promoter chromatin differences. However, DNA hypomethylation extending from unmethylated CpG-rich promoters to immediate upstream and downstream regions was often seen in tissues with the strongest expression of the gene. Occasionally active intragenic promoters were associated with expression of novel ncRNA genes that had a different tissue specificity than that of the host protein-coding gene. Lastly, not only were *KLHL* intragenic and intergenic regions associated with tissue-specific expression, but also, several *KLHL* family genes gave epigenomic/transcriptomic evidence for intragenic and intergenic enhancers in or near adjacent genes exerting *cis* effects on transcription of their neighbors.

## Figures and Tables

**Table 1:** KLHL family genes that are preferentially expressed in skeletal muscle ^a^.

Description	TPM in SkM ^b^	TPM Ratio: SkM to Median of Other Tissues	FPKM in Myoblasts	FPKM Ratio: Myoblasts to Median of Heterologous Cell Cultures ^c^	FPKM Ratio: Myotubes to Myoblasts
*KBTBD12* (Figure S4)	15	13	0.3	0	2.8
*KBTBD13*^d^ (Figure S6)	1.5	25	0	0	0
*KEAP1* (Figure 3)	88	2.4	32	0.7	1.0
*KLHL21* (Figure S10)	84	3.3	31	0.6	1.4
*KLHL30* (Figure 2)	110	145	16	18.0	2.8
*KLHL31* (Figure S2)	18	59	5.6	5.8	872
*KLHL33* (Figure S1)	11	24	0	0	0
*KLHL34* (Figure S3)	8.5	113	0	0	0
*KLHL38* (Figure 2)	90	405	0.1	0	19
*KLHL40* (Figure 1; Figure S5)	303	3373	7.7	#DIV/0!	17
*KLHL41* (Figure 1)	3420	1946	104	1613	254

^a^ We define preferential expression as genes that display a median TPM ≥ 1 in skeletal muscle (SkM) and a ratio of TPM in SkM to the median TPM in 51 other tissues > 3, the only exception is *KEAP1*. TPM, transcripts per kilobase million; FPKM, fragments per kilobase million (like TPM). ^b^ The RNA-seq expression data for SkM and 51 other tissues (Supplemental Table S2) are from the GTEx Analysis Release V8 [22,23]. ^c^ FPKM, fragments per kilobase of exon per million reads mapped, are from technical duplicates from the ENCODE database [24] (Supplemental Table S2). ^d^ Highest expression is in tibial artery (TPM, 2.6).

**Table 2:** *KLHL* family genes that are preferentially expressed in brain ^a^.

Description	Brain Tissue (Median TPM)			Ratio of Brain Tissue TPM to Median TPM of Non-Brain Tissues ^b^		
	Cortex ^c^	Hippoc. ^c^	Cerebel. ^c^	Cortex	Hippoc.	Cerebel.
*ENC1* (Figure 3)	322	87	4.3	56	15	0.7
*KBTBD11* (Figure 4)	59	27	46	17	7.2	12
*KLHL2* (Figure S9)	47	47	21	3.2	45	39
*KLHL32* (Figure 4)	10	12	11	37	3.9	6.0
*KLHL35*^d^ (Figure S9)	3.3	1.6	2.5	8.0	13	2.9
*KLHL4* (Figure S9)	3.6	4.5	1.0	11	15	0.7

^a^ Criterion for preferential expression is that the ratio of TPM in brain frontal cortex to median TPM of non-brain tissues > 3. ^b^ In the GTEx dataset there are 40 non-brain tissues. ^c^ Cortex, frontal cortex; Hippoc, hippocampus; cerebel, cerebellum. ^d^ Highest expression is in testis (TPM, 10.3).

**Table 3:** Epigenetic patterns associated with tissue-specific expression of *KLHL* genes in skeletal muscle or brain ^a^.

Epigenetic Profile Specifically Associated with SkM or Brain	Genes Preferentially Expressed in SkM (Some Also in Heart)	Genes Preferentially Expressed in Brain
Promoter chromatin upstream of TSS ^b^	*KLHL31*, *33*, *34*	None
Promoter chromatin dnstrm of TSS ^b^	*KLHL30*, *31*, *33*, *38*, *41*	*KLHL32*, *35*, *KBTBD11*
Broadening of a constitutively unmethylated region at the TSS	*KLHL21*, *33*, *34*, *40; KBTBD12*	*KLHL2*, *4*, *32* (neurons ^c^); *ENC1*, *KBTBD11* (neurons/fetal brain)
DNA hypomethylation upstream of TSS ^b^	*KLHL30*, *31*, *33*, *34*, *38*, *40*, *KBTBD13*	*KLHL32* (neurons)
DNA hypomethylation dnstrm of TSS ^b^	*KLHL21*, *30*, *31*, *33*, *41; KBTBD12*	*KLHL2*, *4*, *32* (neurons)
Super-enhancer	*KLHL 21*, *30*, *31*, *38*, *40*, *41*	*ENC1*
Intragenic enhancer chromatin	*KLHL21*, *30*, *31*, *32*, *33*, *34*, *38*, *40*, *41; KEAP1; KBTBD12*, *13*	*KLHL2*, *35; KBTBD11*, *ENC1*
Gene-upstream enhancer chromatin	*KLHL21*, *30*, *31*, *33*, *34*, *38*, *40*, *41*	*KLHL32*, *KBTBD11*, *ENC1* (neurons/fetal brain)
Gene-downstream enhancer chromatin	*KLHL21*, *38*, *KBTBD12*	*KBTBD11*, *ENC1* (fetal brain)
DNA hypomethylation in enhancer chromatin	*KLHL21*, *30*, *31*, *33*, *34*, *38*, *40*, *41*, *KBTBD12*, *13*	*KLHL2* (hippocampus, anterior caudate)

^a^ The epigenetic features for the listed *KLHL* and *KBTBD* genes are shown in Figure 1, Figure 2, Figure 3 and Figure 4 and Figures S1–S6, S8–S10. Only tissue-specific epigenetic features that correlated with preferential expression in skeletal muscle (SkM; sometimes also in heart) or in brain are shown. TSS, transcription start site; constitutive unmethylated region, mostly or completely unmethylated in all or almost all of the >15 examined tissues; ^b^ promoter chromatin or DNA hypomethylation immediately upstream or downstream of the TSS; ^c^ enhancer and promoter chromatin states are not available for neurons.

## Supplementary Materials

Supplemental files can be found at https://www.mdpi.com/1422-0067/21/21/8394/s1.

## Abbreviations

BS	bisulfite
CAGE	5′ Cap Analysis of Gene Expression
CGI	CpG island
ChIP-seq	chromatin immunoprecipitation with next-gen sequencing
CTCF	CCCTC-binding factor
Enh	enhancer
FPKM	fragments per kilobase of exon per million reads mapped
H3K27ac	Histone H3 lysine 27 acetylation
H3K4me1	histone H3 lysine 4 monomethylation
H3K4me3	histone H3 lysine 4 trimethylation
LCL	lymphoblastoid cell line
LMR	low methylated region
Micro-C	a high-resolution type of chromatin capture analysis
ncRNA	non-coding RNA
NHLF	normal human lung fibroblasts
ORF	open reading frame
PBMC	peripheral blood mononuclear cells
Prom	promoter
SkM	skeletal muscle
TAD	topologically associating domain
TPM	transcripts per kilobase million
TSS	transcription start site
Txn	transcription

## References

[1] Guo Y., Yu S., Zhang C., Kong A.N. (2015). Epigenetic regulation of Keap1-Nrf2 signaling. Free Radic. Biol. Med..

[2] Varshavsky A. (2017). The Ubiquitin System, Autophagy, and Regulated Protein Degradation. Annu. Rev. Biochem..

[3] Mumtaz P.T., Taban Q., Dar M.A., Mir S., Haq Z.U., Zargar S.M., Shah R.A., Ahmad S.M. (2020). Deep Insights in Circular RNAs: From biogenesis to therapeutics. Biol. Proced. Online.

[4] Kundaje A., Meuleman W., Ernst J., Bilenky M., Yen A., Heravi-Moussavi A., Kheradpour P., Zhang Z., Wang J., Roadmap Epigenomics Consortium (2015). Integrative analysis of 111 reference human epigenomes. Nature.

[5] Dhanoa B.S., Cogliati T., Satish A.G., Bruford E.A., Friedman J.S. (2013). Update on the Kelch-like (KLHL) gene family. Hum. Genom..

[6] Shi X., Xiang S., Cao J., Zhu H., Yang B., He Q., Ying M. (2019). Kelch-like proteins: Physiological functions and relationships with diseases. Pharm. Res..

[7] Gupta V.A., Beggs A.H. (2014). Kelch proteins: Emerging roles in skeletal muscle development and diseases. Skelet. Muscle.

[8] Elshaer M., ElManawy A.I., Hammad A., Namani A., Wang X.J., Tang X. (2020). Integrated data analysis reveals significant associations of KEAP1 mutations with DNA methylation alterations in lung adenocarcinomas. Aging (Albany NY).

[9] Sewry C.A., Laitila J.M., Wallgren-Pettersson C. (2019). Nemaline myopathies: A current view. J. Muscle Res. Cell Motil..

[10] Jirka C., Pak J.H., Grosgogeat C.A., Marchetii M.M., Gupta V.A. (2019). Dysregulation of NRAP degradation by KLHL41 contributes to pathophysiology in Nemaline Myopathy. Hum. Mol. Genet..

[11] Zhang Z., Turer E., Li X., Zhan X., Choi M., Tang M., Press A., Smith S.R., Divoux A., Moresco E.M. (2016). Insulin resistance and diabetes caused by genetic or diet-induced KBTBD2 deficiency in mice. Proc. Natl. Acad. Sci. USA.

[12] Yoshida S., Araki Y., Mori T., Sasaki E., Kasagi Y., Isobe K., Susa K., Inoue Y., Bomont P., Okado T. (2018). Decreased KLHL3 expression is involved in the pathogenesis of pseudohypoaldosteronism type II caused by cullin 3 mutation in vivo. Clin. Exp. Nephrol..

[13] Hedberg-Oldfors C., Abramsson A., Osborn D.P.S., Danielsson O., Fazlinezhad A., Nilipour Y., Hubbert L., Nennesmo I., Visuttijai K., Bharj J. (2019). Cardiomyopathy with lethal arrhythmias associated with inactivation of KLHL24. Hum. Mol. Genet..

[14] Deaton A.M., Bird A. (2011). CpG islands and the regulation of transcription. Genes Dev..

[15] Ehrlich K.C., Paterson H.L., Lacey M., Ehrlich M. (2016). DNA hypomethylation in intragenic and intergenic enhancer chromatin of muscle-specific genes usually correlates with their expression. Yale J. Biol. Med..

[16] Ehrlich K.C., Lacey M., Ehrlich M. (2020). Epigenetics of Skeletal Muscle-Associated Genes in the ASB, LRRC, TMEM, and OSBPL Gene Families. Epigenomes.

[17] Heberle E., Bardet A.F. (2019). Sensitivity of transcription factors to DNA methylation. Essays Biochem..

[18] Lazaris C., Aifantis I., Tsirigos A. (2020). On Epigenetic Plasticity and Genome Topology. Trends Cancer.

[19] GTEx_Consortium (2015). Human genomics. The Genotype-Tissue Expression (GTEx) pilot analysis: Multitissue gene regulation in humans. Science.

[20] Illingworth R.S., Gruenewald-Schneider U., De Sousa D., Webb S., Merusi C., Kerr A.R., James K.D., Smith C., Walker R., Andrews R. (2015). Inter-individual variability contrasts with regional homogeneity in the human brain DNA methylome. Nucleic Acids Res..

[21] Dayalan Naidu S., Dinkova-Kostova A.T. (2020). KEAP1, a cysteine-based sensor and a drug target for the prevention and treatment of chronic disease. Open Biol..

[22] Pirinen M., Lappalainen T., Zaitlen N.A., Dermitzakis E.T., Donnelly P., McCarthy M.I., Rivas M.A., GTEx_Consortium (2015). Assessing allele-specific expression across multiple tissues from RNA-seq read data. Bioinformatics.

[23] Searle B.C., Gittelman R.M., Manor O., Akey J.M. (2016). Detecting Sources of Transcriptional Heterogeneity in Large-Scale RNA-Seq Data Sets. Genetics.

[24] Haeussler M., Zweig A.S., Tyner C., Speir M.L., Rosenbloom K.R., Raney B.J., Lee C.M., Lee B.T., Hinrichs A.S., Gonzalez J.N. (2019). The UCSC Genome Browser database: 2019 update. Nucleic Acids Res..

[25] Gheorghe M., Sandve G.K., Khan A., Cheneby J., Ballester B., Mathelier A. (2019). A map of direct TF-DNA interactions in the human genome. Nucleic Acids Res..

[26] Xi H., Shulha H.P., Lin J.M., Vales T.R., Fu Y., Bodine D.M., McKay R.D., Chenoweth J.G., Tesar P.J., Furey T.S. (2007). Identification and characterization of cell type-specific and ubiquitous chromatin regulatory structures in the human genome. PLoS Genet..

[27] Whyte W.A., Orlando D.A., Hnisz D., Abraham B.J., Lin C.Y., Kagey M.H., Rahl P.B., Lee T.I., Young R.A. (2013). Master transcription factors and mediator establish super-enhancers at key cell identity genes. Cell.

[28] Khan A., Zhang X. (2016). dbSUPER: A database of super-enhancers in mouse and human genome. Nucleic Acids Res..

[29] Blondelle J., Tallapaka K., Seto J.T., Ghassemian M., Clark M., Laitila J.M., Bournazos A., Singer J.D., Lange S. (2019). Cullin-3 dependent deregulation of ACTN1 represents a new pathogenic mechanism in nemaline myopathy. JCI Insight.

[30] Bowlin K.M., Embree L.J., Garry M.G., Garry D.J., Shi X. (2013). Kbtbd5 is regulated by MyoD and restricted to the myogenic lineage. Differentiation.

[31] Cao Y., Yao Z., Sarkar D., Lawrence M., Sanchez G.J., Parker M.H., MacQuarrie K.L., Davison J., Morgan M.T., Ruzzo W.L. (2010). Genome-wide MyoD binding in skeletal muscle cells: A potential for broad cellular reprogramming. Dev. Cell.

[32] Hsieh T.S., Fudenberg G., Goloborodko A., Rando O.J. (2016). Micro-C XL: Assaying chromosome conformation from the nucleosome to the entire genome. Nat. Methods.

[33] Van B., Nishi M., Komazaki S., Ichimura A., Kakizawa S., Nakanaga K., Aoki J., Park K.H., Ma J., Ueyama T. (2015). Mitsugumin 56 (hedgehog acyltransferase-like) is a sarcoplasmic reticulum-resident protein essential for postnatal muscle maturation. FEBS Lett..

[34] Zhang P., Zhang L., Li Y., Zhu S., Zhao M., Ding S., Li J. (2018). Quantitative Proteomic Analysis To Identify Differentially Expressed Proteins in Myocardium of Epilepsy Using iTRAQ Coupled with Nano-LC-MS/MS. J. Proteome Res..

[35] de Winter J.M., Molenaar J.P., Yuen M., van der Pijl R., Shen S., Conijn S., van de Locht M., Willigenburg M., Bogaards S.J., van Kleef E.S. (2020). KBTBD13 is an actin-binding protein that modulates muscle kinetics. J. Clin. Investig..

[36] Sartorelli V., Lauberth S.M. (2020). Enhancer RNAs are an important regulatory layer of the epigenome. Nat. Struct. Mol. Biol..

[37] Bodine S.C., Baehr L.M. (2014). Skeletal muscle atrophy and the E3 ubiquitin ligases MuRF1 and MAFbx/atrogin-1. Am. J. Physiol. Endocrinol. Metab..

[38] de O’Coelho P., Guarnier F.A., Figueiredo L.B., Zaramela L.S., Pacini E.S.A., Godinho R.O., Gomes M.D. (2019). Identification of potential target genes associated with the reversion of androgen-dependent skeletal muscle atrophy. Arch. Biochem. Biophys..

[39] Baertsch R., Diekhans M., Kent W.J., Haussler D., Brosius J. (2008). Retrocopy contributions to the evolution of the human genome. BMC Genom..

[40] Sukari A., Muqbil I., Mohammad R.M., Philip P.A., Azmi A.S. (2016). F-BOX proteins in cancer cachexia and muscle wasting: Emerging regulators and therapeutic opportunities. Semin. Cancer Biol..

[41] Bellezza I., Giambanco I., Minelli A., Donato R. (2018). Nrf2-Keap1 signaling in oxidative and reductive stress. Biochim. Biophys. Acta Mol. Cell Res..

[42] van Rooij E., Quiat D., Johnson B.A., Sutherland L.B., Qi X., Richardson J.A., Kelm R.J., Olson E.N. (2009). A family of microRNAs encoded by myosin genes governs myosin expression and muscle performance. Dev. Cell.

[43] Wang X.J., Zhang D.D. (2009). Ectodermal-neural cortex 1 down-regulates Nrf2 at the translational level. PLoS ONE.

[44] Mesman S., Kruse S.J., Smidt M.P. (2018). Expression analyzes of early factors in midbrain differentiation programs. Gene Expr. Patterns.

[45] Lister R., Mukamel E.A., Nery J.R., Urich M., Puddifoot C.A., Johnson N.D., Lucero J., Huang Y., Dwork A.J., Schultz M.D. (2013). Global epigenomic reconfiguration during mammalian brain development. Science.

[46] White C.C., Yang H.S., Yu L., Chibnik L.B., Dawe R.J., Yang J., Klein H.U., Felsky D., Ramos-Miguel A., Arfanakis K. (2017). Identification of genes associated with dissociation of cognitive performance and neuropathological burden: Multistep analysis of genetic, epigenetic, and transcriptional data. PLoS Med..

[47] van den Oord E.J., Clark S.L., Xie L.Y., Shabalin A.A., Dozmorov M.G., Kumar G., Swedish Schizophrenia C., Vladimirov V.I., Magnusson P.K., Aberg K.A. (2016). A Whole Methylome CpG-SNP Association Study of Psychosis in Blood and Brain Tissue. Schizophr. Bull..

[48] Terragni J., Zhang G., Sun Z., Pradhan S., Song L., Crawford G.E., Lacey M., Ehrlich M. (2014). Notch signaling genes: Myogenic DNA hypomethylation and 5-hydroxymethylcytosine. Epigenetics.

[49] Do C., Lang C.F., Lin J., Darbary H., Krupska I., Gaba A., Petukhova L., Vonsattel J.P., Gallagher M.P., Goland R.S. (2016). Mechanisms and Disease Associations of Haplotype-Dependent Allele-Specific DNA Methylation. Am. J. Hum. Genet..

[50] Worton L.E., Shi Y.C., Smith E.J., Barry S.C., Gonda T.J., Whitehead J.P., Gardiner E.M. (2017). Ectodermal-Neural Cortex 1 Isoforms Have Contrasting Effects on MC3T3-E1 Osteoblast Mineralization and Gene Expression. J. Cell Biochem..

[51] Watanabe K., Yokota K., Yoshida K., Matsumoto A., Iwamoto S. (2019). Kbtbd11 contributes to adipocyte homeostasis through the activation of upstream stimulatory factor 1. Heliyon.

[52] Kachroo P., Szymczak S., Heinsen F.A., Forster M., Bethune J., Hemmrich-Stanisak G., Baker L., Schrappe M., Stanulla M., Franke A. (2018). NGS-based methylation profiling differentiates TCF3-HLF and TCF3-PBX1 positive B-cell acute lymphoblastic leukemia. Epigenomics.

[53] Szabo Q., Bantignies F., Cavalli G. (2019). Principles of genome folding into topologically associating domains. Sci. Adv..

[54] Andersson R., Sandelin A. (2020). Determinants of enhancer and promoter activities of regulatory elements. Nat. Rev. Genet..

[55] Chen H., Xiao J., Shao T., Wang L., Bai J., Lin X., Ding N., Qu Y., Tian Y., Chen X. (2019). Landscape of Enhancer-Enhancer Cooperative Regulation during Human Cardiac Commitment. Mol. Ther. Nucleic Acids.

[56] Chen Q., Li C., Gong Z., Chun Yong Chan E., Snyder S.A., Lam S.H. (2017). Common deregulated gene expression profiles and morphological changes in developing zebrafish larvae exposed to environmental-relevant high to low concentrations of glucocorticoids. Chemosphere.

[57] Papizan J.B., Garry G.A., Brezprozvannaya S., McAnally J.R., Bassel-Duby R., Liu N., Olson E.N. (2017). Deficiency in Kelch protein Klhl31 causes congenital myopathy in mice. J. Clin. Investig..

[58] Wang X., Cairns M.J., Yan J. (2019). Super-enhancers in transcriptional regulation and genome organization. Nucleic Acids Res..

[59] Bell E., Curry E.W., Megchelenbrink W., Jouneau L., Brochard V., Tomaz R.A., Mau K.H.T., Atlasi Y., de Souza R.A., Marks H. (2020). Dynamic CpG methylation delineates subregions within super-enhancers selectively decommissioned at the exit from naive pluripotency. Nat. Commun..

[60] Jeziorska D.M., Murray R.J.S., De Gobbi M., Gaentzsch R., Garrick D., Ayyub H., Chen T., Li E., Telenius J., Lynch M. (2017). DNA methylation of intragenic CpG islands depends on their transcriptional activity during differentiation and disease. Proc. Natl. Acad. Sci. USA.

[61] Ponnaluri V.K., Ehrlich K.C., Zhang G., Lacey M., Johnston D., Pradhan S., Ehrlich M. (2017). Association of 5-hydroxymethylation and 5-methylation of DNA cytosine with tissue-specific gene expression. Epigenetics.

[62] Ehrlich M. (2019). DNA hypermethylation in disease: Mechanisms and clinical relevance. Epigenetics.

[63] Paxton C.W., Cosgrove R.A., Drozd A.C., Wiggins E.L., Woodhouse S., Watson R.A., Spence H.J., Ozanne B.W., Pell J.M. (2011). BTB-Kelch protein Krp1 regulates proliferation and differentiation of myoblasts. Am. J. Physiol. Cell Physiol..

[64] Xian S., Li J., Zhang Z. (2020). miR-26b inhibits isoproterenol-induced cardiac fibrosis via the Keap1/Nrf2 signaling pathway. Exp. Ther. Med..

[65] Zhou Y., Tang X., Niu L., Liu Y., Wang B., He J. (2020). Ectodermal-neural cortex 1 as a novel biomarker predicts poor prognosis and induces metastasis in breast cancer by promoting Wnt/beta-catenin pathway. J. Cell Mol. Med..

[66] Watanabe K., Yoshida K., Iwamoto S. (2019). Kbtbd11 gene expression in adipose tissue increases in response to feeding and affects adipocyte differentiation. J. Diabetes Investig..

[67] Li W., Pung D., Su Z.Y., Guo Y., Zhang C., Yang A.Y., Zheng X., Du Z.Y., Zhang K., Kong A.N. (2016). Epigenetics Reactivation of Nrf2 in Prostate TRAMP C1 Cells by Curcumin Analogue FN1. Chem. Res. Toxicol..

[68] Fabrizio F.P., Sparaneo A., Centra F., Trombetta D., Storlazzi C.T., Graziano P., Maiello E., Fazio V.M., Muscarella L.A. (2019). Methylation Density Pattern of KEAP1 Gene in Lung Cancer Cell Lines Detected by Quantitative Methylation Specific PCR and Pyrosequencing. Int. J. Mol. Sci..

[69] Trapnell C., Roberts A., Goff L., Pertea G., Kim D., Kelley D.R., Pimentel H., Salzberg S.L., Rinn J.L., Pachter L. (2012). Differential gene and transcript expression analysis of RNA-seq experiments with TopHat and Cufflinks. Nat. Protoc..

[70] Tsumagari K., Baribault C., Terragni J., Varley K.E., Gertz J., Pradhan S., Badoo M., Crain C.M., Song L., Crawford G.E. (2013). Early de novo DNA methylation and prolonged demethylation in the muscle lineage. Epigenetics.

[71] Chen P.H., Smith T.J., Wu J., Siesser P.F., Bisnett B.J., Khan F., Hogue M., Soderblom E., Tang F., Marks J.R. (2017). Glycosylation of KEAP1 links nutrient sensing to redox stress signaling. EMBO J..

[72] Song Q., Decato B., Hong E.E., Zhou M., Fang F., Qu J., Garvin T., Kessler M., Zhou J., Smith A.D. (2013). A reference methylome database and analysis pipeline to facilitate integrative and comparative epigenomics. PLoS ONE.

[73] Krietenstein N., Abraham S., Venev S.V., Abdennur N., Gibcus J., Hsieh T.S., Parsi K.M., Yang L., Maehr R., Mirny L.A. (2020). Ultrastructural Details of Mammalian Chromosome Architecture. Mol. Cell.

[74] Papadopoulos J.S., Agarwala R. (2007). COBALT: Constraint-based alignment tool for multiple protein sequences. Bioinformatics.

