# Supplementary material for: Epigenetics of Muscle- and Brain-Specific Expression of KLHL Family Genes
Source: Int J Mol Sci. 2020 Nov 9;21(21):8394. doi: 10.3390/ijms21218394 (PMC7672584; doi:10.3390/ijms21218394)
Supplement: Supplementary file 1 [file ijms-21-08394-s001.zip › Ehrlich_KLHL_Epigen_SupplFigS1-S10-KLHL_RevF_10_30_20.pdf]

Epigenetics of muscle- and brain-specific expression of KLHL family genes

Kenneth C. Ehrlich, Carl Baribault, Melanie Ehrlich

Supplemental Figures

Figure S1. Preferential expression in skeletal muscle of the little-studied gene, *KLHL33* is associated with spreading of DNA hypomethylation from a CpG island at the transcription start site

Figure S2. Preferential expression in heart and skeletal muscle of *KLHL31*, a gene implicated in congenital myopathy, is accompanied by tissue-specific promoter and intragenic DNA hypomethylation

Figure S3. Preferential expression and enhancer chromatin in skeletal muscle and heart at *KLHL34*, a little studied gene, and its upstream neighbor *SMPX* (small muscle protein X-linked gene)

Figure S4. *KBTBD12* has an intragenic enhancer chromatin region that is hypomethylated in intron 4 that probably contributes to its preferential expression in skeletal muscle and heart

Figure S5. *KLHL40* and surrounding genes can share a topologically associating domain (TAD)

Figure S6. The nemaline myopathy gene *KBTBD13*, has low, but preferential, expression in skeletal muscle accompanied by tissue-specific epigenetic marks upstream of the gene and in the single-exon gene body

Figure S7. Phylogenetic tree of full-length proteins encoded by *KLHL* and *KBTBD* genes

Figure S8. *KLHL38* and *FBXO32* can share a topologically associating domain

Figure S9. Epigenetics of three additional *KLHL* genes preferentially expressed in brain (*KLHL2, KLHL4* and *KLHL35)*

Figure S10. *KLHL21,* a gene required for cytokinesis, is widely expressed although with highest expression in skeletal muscle and has the most DNA hypomethylation and enhancer chromatin in skeletal muscle

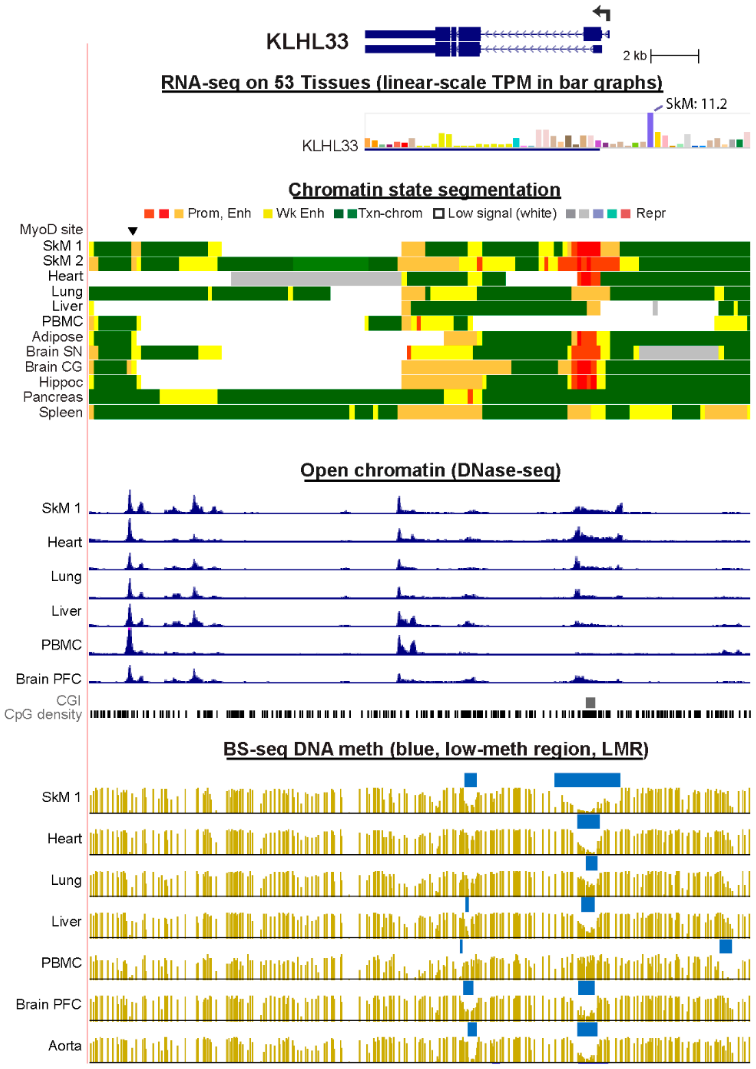

Figure S1. Preferential expression in skeletal muscle of the little-studied gene *KLHL33* is associated with spreading of DNA hypomethylation from a CpG island at the transcription start site. All tracks are from the UCSC Genome Browser, hg19 (http://genome.ucsc.edu). Coordinates for this gene region are chr14:20,882,384-20,910,286. The GTEx RNA-seq expression profile in this and subsequent figures is shown as bar graphs with linearly displayed median values for TPM unless otherwise indicated (https://gtexportal.org/home/). Chromatin state segmentation shows the 18-state profiles from the Roadmap Epigenetics Project (see Methods). Open chromatin was determined by DNase hypersensitivity (Roadmap). Bisulfite-seq (BS-seq) tracks have blue bars indicating regions of low-methylation (LMRs) relative to methylation in the same tissue or cell-type. The gray box in the CpG density track indicates a CpG island (CGI). This CGI is referred to in Table 3 as constitutively unmethylated; from >15 different tissue types examined, the only ones with appreciable methylation in this region are blood samples or tissues that contain especially much blood. TPM, transcripts per kilobase millions; Prom, promoter; Enh, enhancer; Txn, transcription; Repr, repressed; SkM 1, skeletal muscle from psoas; SkM 2, skeletal muscle from leg; PBMC, peripheral blood mononuclear cells; brain PFC, prefrontal cortex; brain SN, substantia nigra; brain CG, cingulate gyrus; Hippoc, hippocampus.

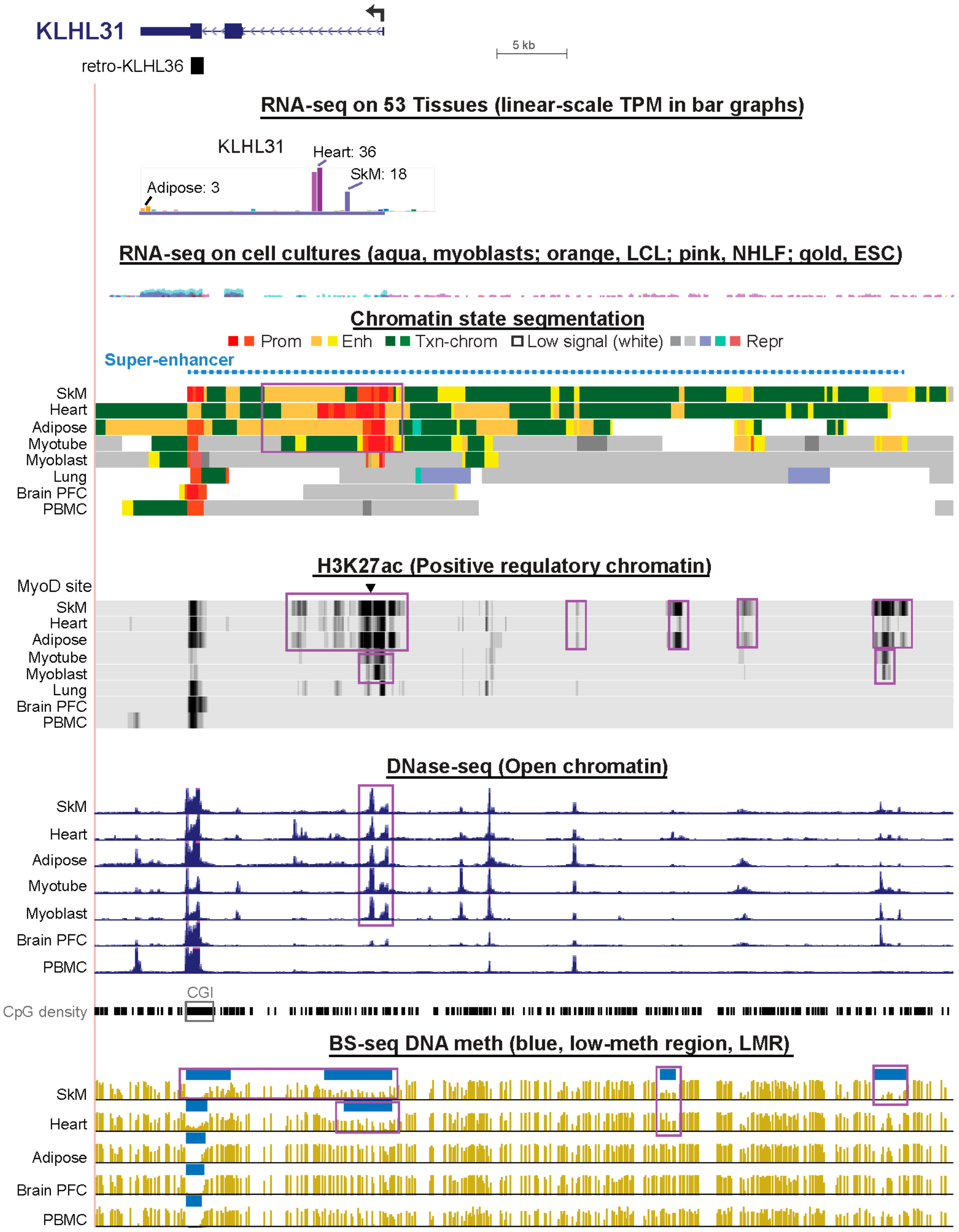

Figure S2. Preferential expression in heart and skeletal muscle of *KLHL31*, a gene implicated in congenital myopathy, is accompanied by tissue-specific promoter and intragenic DNA hypomethylation. Tracks for this gene region (chr6:53,509,089-53,571,737) are as described in the legend to Figure S1. An extended upstream region is shown to indicate regions of epigenetic marks positively associated with tissue-specific expression. Purple boxes, tissue-specific epigenetic marks of particular interest for their association with tissue-specific expression patterns. H3K27ac, histone H3 lysine-27 acetylation, a mark generally seen in active promoter or enhancer regions.

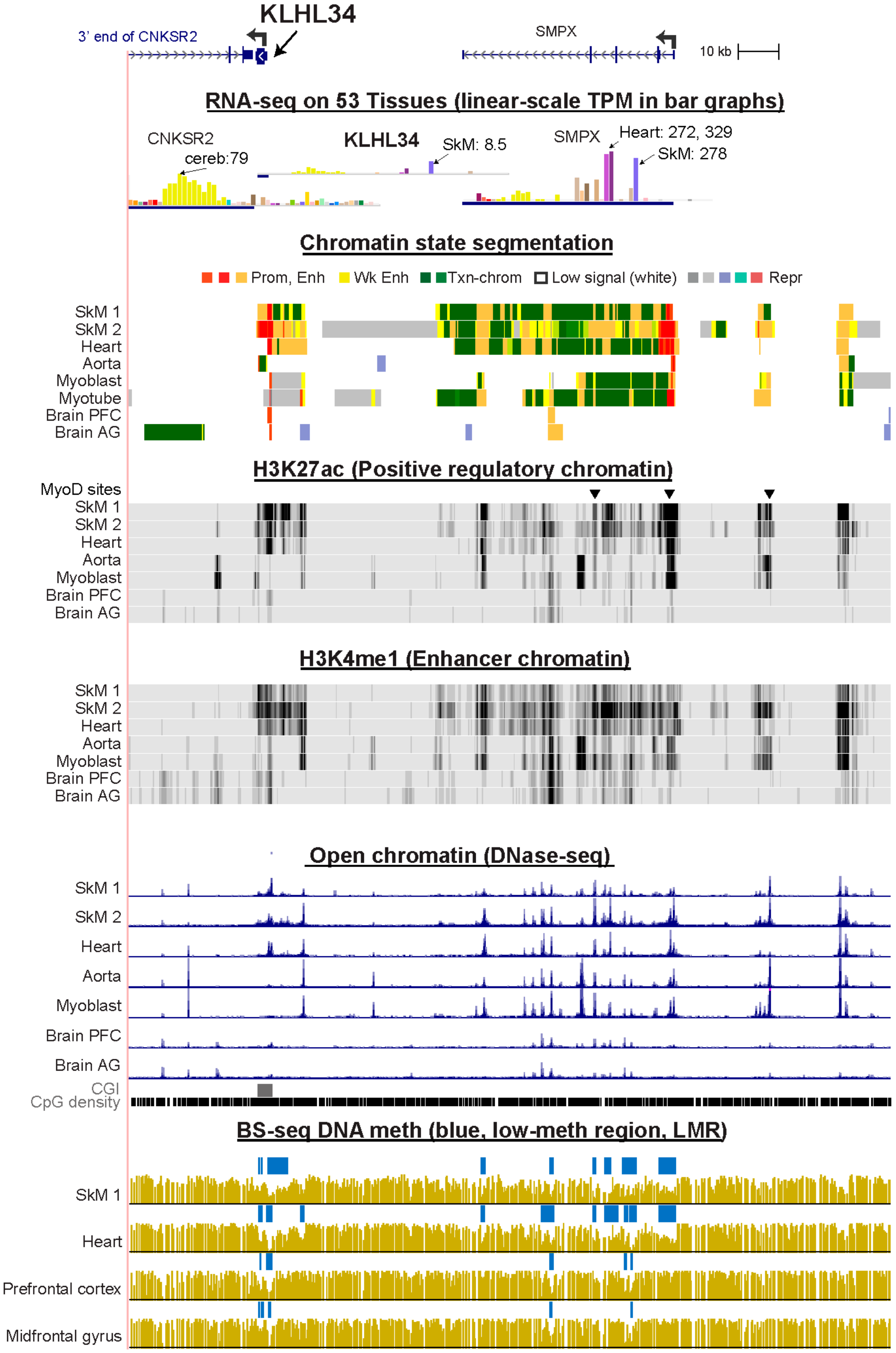

Figure S3. Preferential expression and enhancer chromatin in skeletal muscle and heart at *KLHL34*, a little studied gene, and its upstream neighbor *SMPX* (small muscle protein X-linked gene). Descriptions of tracks for the region shown (chrX:21,641,970-21,829,630) are as in Figure S1. Brain AG, angular gyrus.

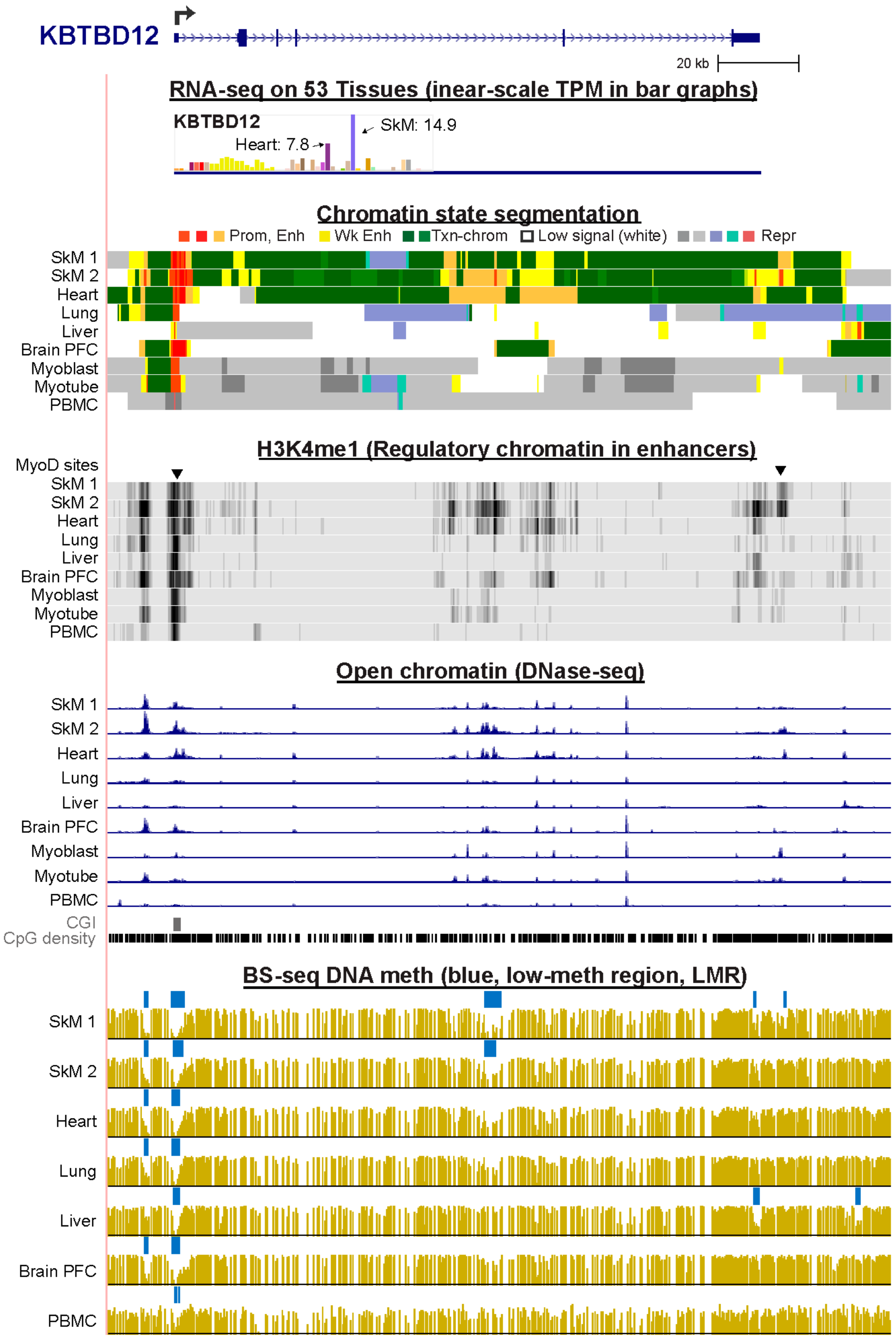

Figure S4. ***KBTBD12*, another example of little-studied *KLHL* family gene, has an intragenic enhancer chromatin region that is hypomethylated in intron 4 that probably contributes to its preferential expression in skeletal muscle and heart.** Tracks for chr3:127,625,748-127,722,667 are as in Figure S2 except that the signal for histone H3 lysine-4 monomethylation (H3K4me1) is shown rather than the signal for H3K27ac. This track was chosen because it is more consistent with the low but observable expression of this gene in tissues other that SkM and heart.

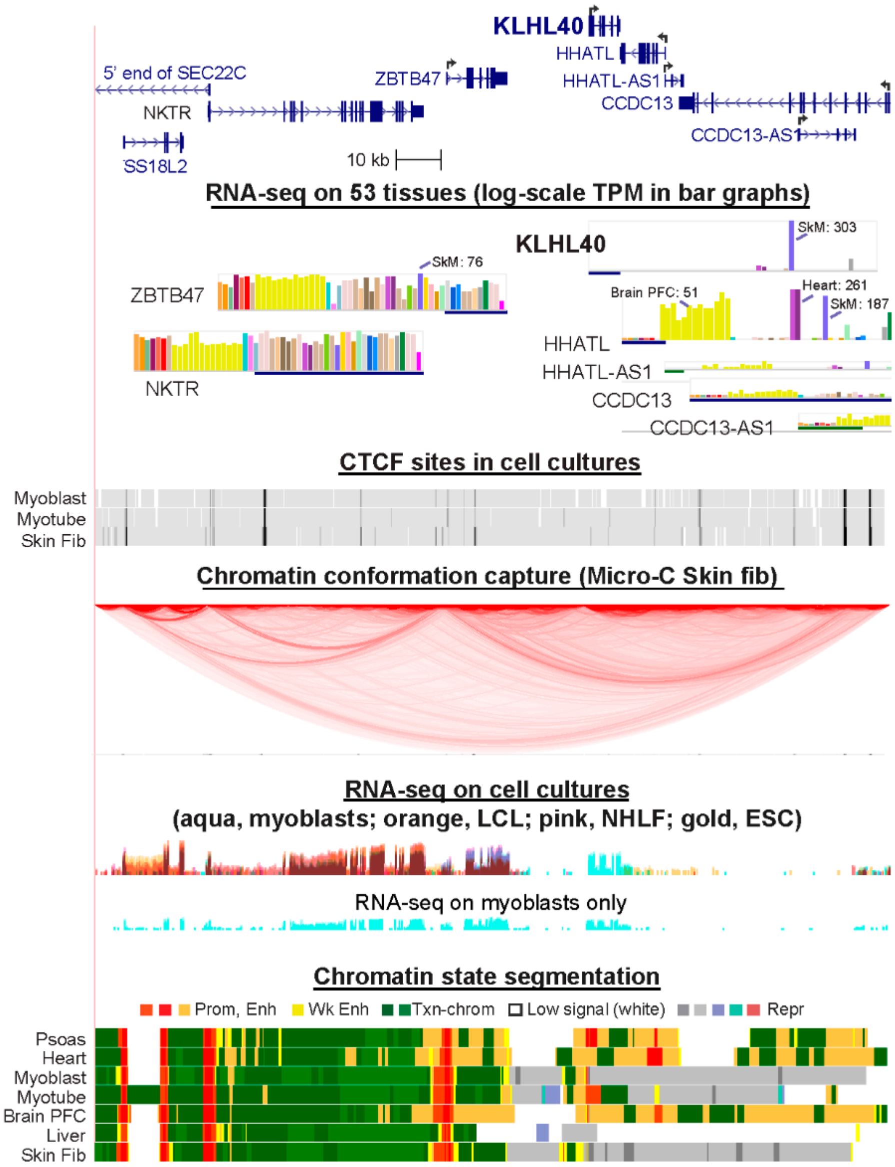

Figure S5. ***KLHL40* and surrounding genes can share a topologically associating domain (TAD).** An expanded region (chr3:42,616,960-42,794,592; 177,633 bp) from that shown in Figure 1E - H. This region includes the neighboring genes *NKTR, ZBTB47*, *HHATL*, and *CCDC13*. Sites for CTCF (CCCTC-binding factor), a transcription factor that often helps define gene boundaries, is shown for myoblasts, myotubes, and skin fibroblasts but is similar in many other cell types. These tracks are from ChIP-seq profiles (Histone Modifications from ENCODE/Broad Institute Track Set, UCSC Genome Browser). TADs are deduced from Micro-C results on HFFc6 foreskin fibroblasts displayed in hg38 at the UCSC Genome Browser and transposed to hg19 coordinates; the intensity of the red color is proportional to the frequency of long-distance interactions. Comparable myoblast TAD profiles are not available. RNA-seq profiling of cell cultures is shown as both overlapping signal from four cell types and separately, for myoblasts (Transcription Levels Assayed by RNA-seq on 9 cell lines from ENCODE data, UCSC Genome Browser). Fib, fibroblasts; LCL, lymphoblastoid cell line GM12878; NHLF, normal human lung fibroblasts; ESC, embryonic stem cells

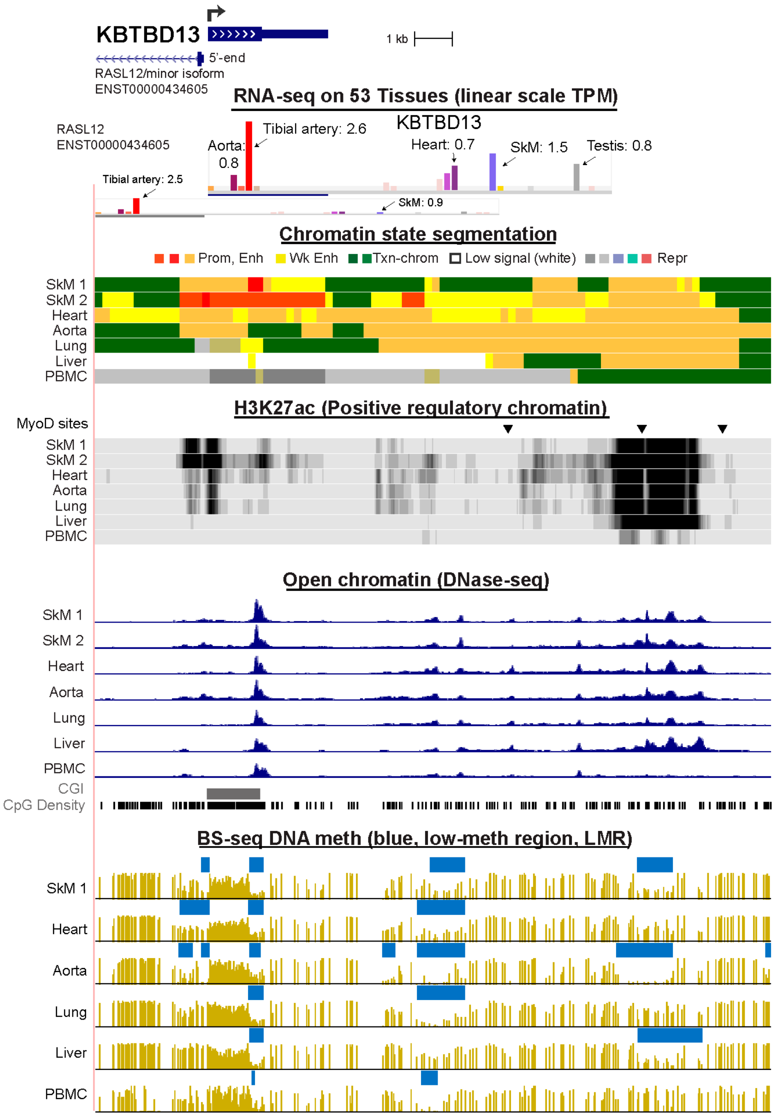

Figure S6. The nemaline myopathy gene ***KBTBD13* has low, but preferential, expression in skeletal muscle accompanied by tissue-specific epigenetic marks upstream of the gene and in the single-exon gene body.** *KBTBD13* exhibits its second highest expression levels in SkM, although it is only expressed at a low level in this tissue. The highest expressing tissue, tibial artery (like testis), does not have available genome-wide epigenetic data. Enhancer chromatin and DNA hypomethylation in the upstream region of this gene observed specifically in SkM, heart, and aorta are consistent with its higher expression in these tissues than in most of the almost 50 other examined tissues. SkM is the only tissue with promoter chromatin at the 5′ end of the gene. However, there is a minor isoform of *RASL12* which shares a bidirectional promoter with *KLHL13* and similar tissue-specificity to that of *KBTBD13*. The major *RASL12* isoforms have their TSS ~9 kb downstream of this isoform's TSS. Tracks and abbreviations are as in the other supplementary figures for coordinates chr15:65,366,190-65,383,838 but the RNA-seq bar graph has different linear scales for *KBTBD13* and this minor *RASL12* isoform.

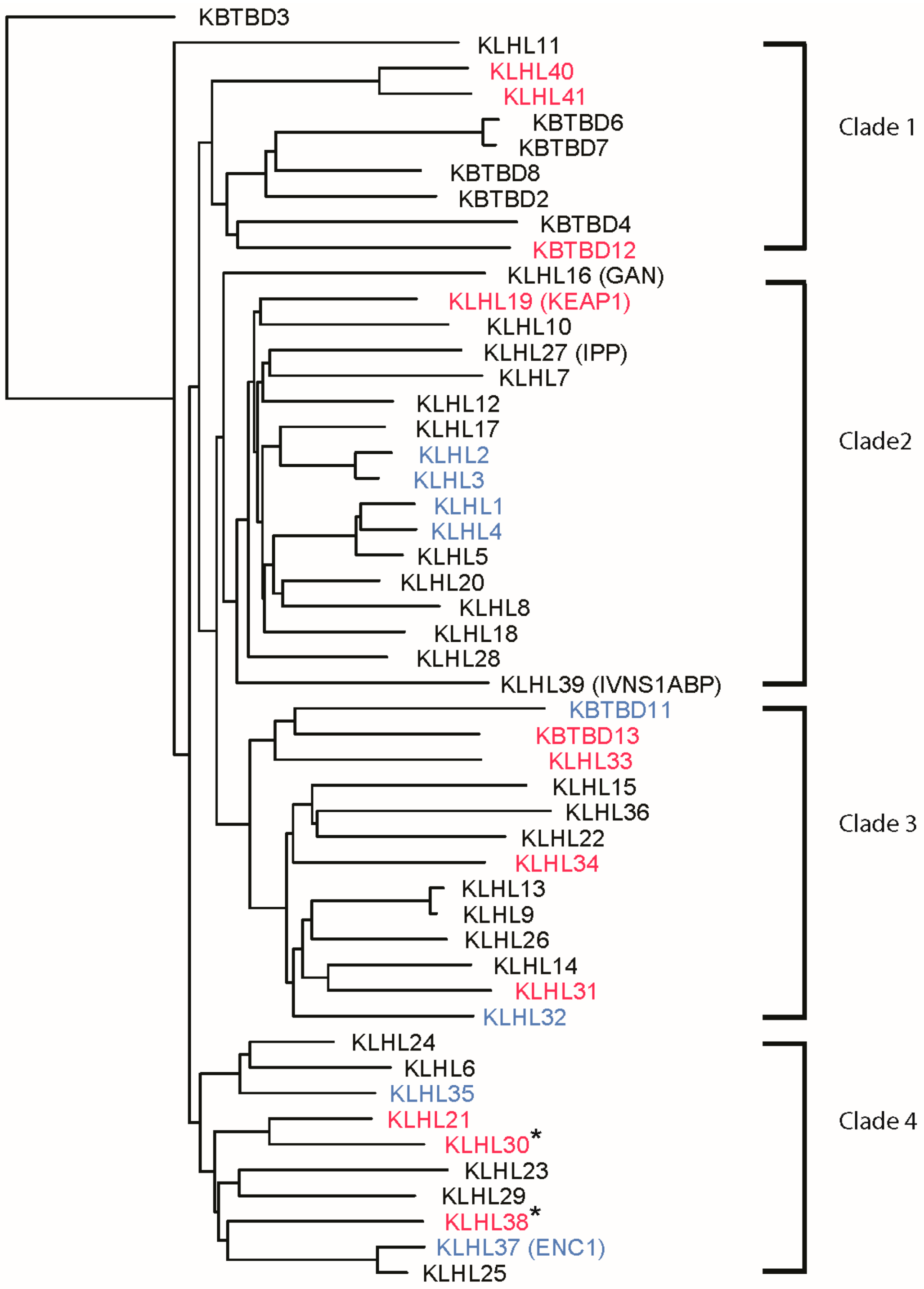

Figure S7. **Phylogenetic tree of encoded full-length proteins encoded by *KLHL* and *KBTBD* family genes**. Only main cladal distributions are indicated. Red font, SkM preferentially expressed genes; blue font, brain preferentially expressed genes including cerebellum-preferentially expressed genes, which were not examined for their epigenetics due to lack of available whole-genome chromatin state profiles. Stars (*) indicate two genes in which retrocopies are in the same clade.

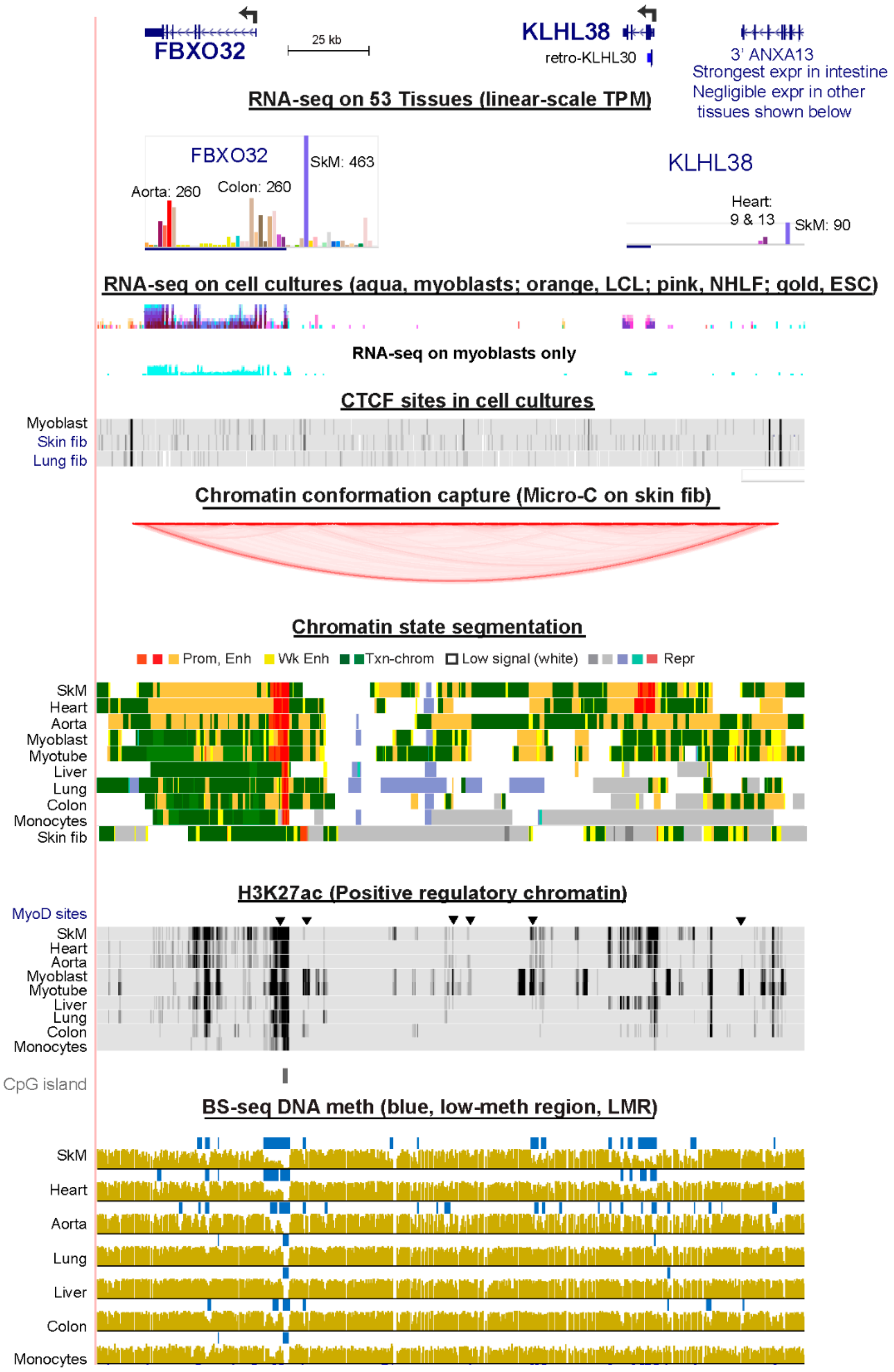

Figure S8. ***KLHL38* and *FBXO32* can share a topologically associating domain**. Region shown is chr8:123,475,406-123,701,369, which is a larger region than that viewed for these two genes in Figure 2E-H. TADs were analyzed in genome assembly hg38 using the Micro-C chromatin conformation capture tool described in Figure S5 and transferred from hg38 to hg19 coordinates. CTCF sites are for two types of fibroblasts and for myoblasts. Other tracks are as previously described.

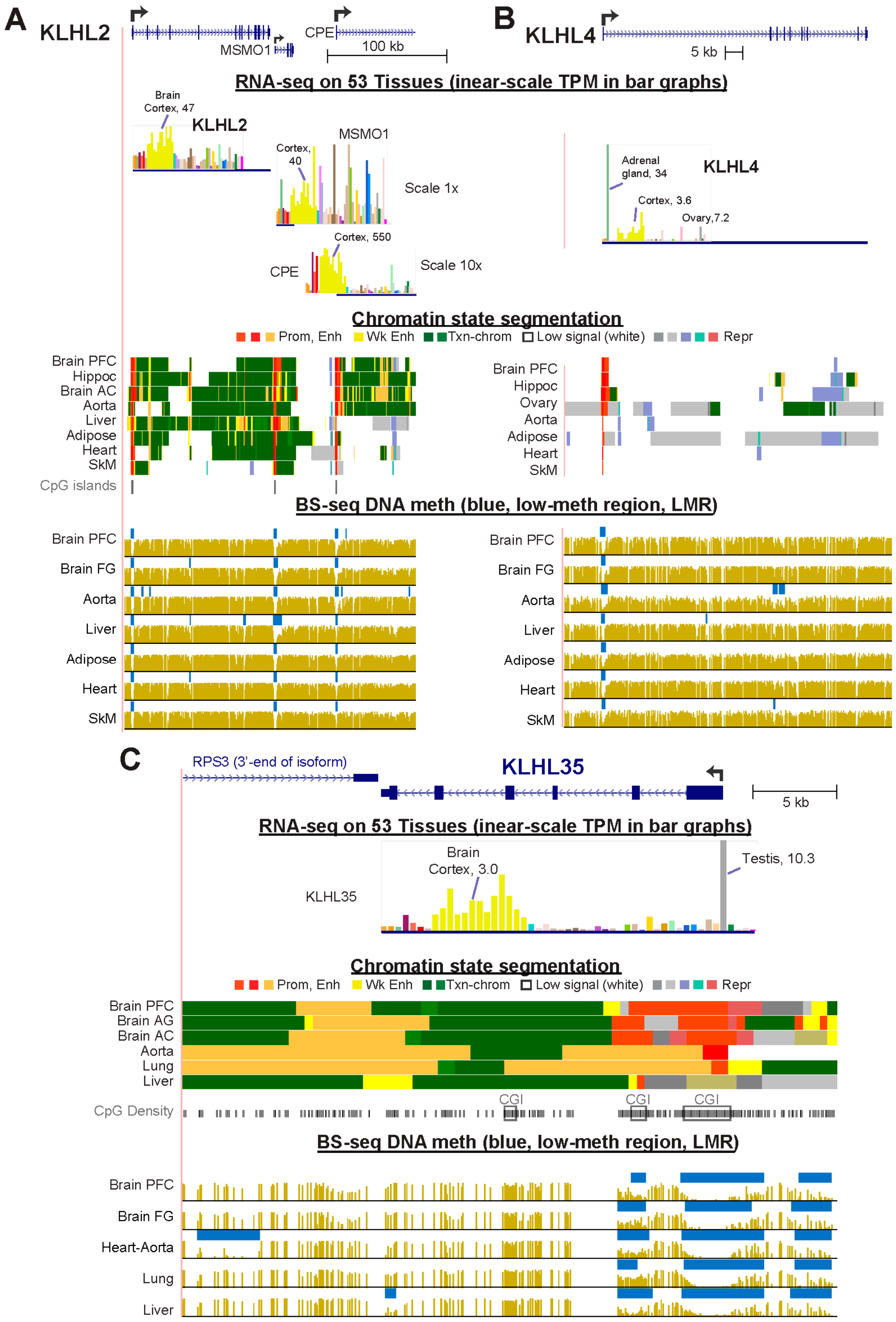

Figure S9. Epigenetics of three additional *KLHL* genes preferentially expressed in brain. (A) *KLHL2* (chr4:166,122,291-166,366,768). **(B)** *KLHL4* (chrX:86,750,470-86,939,163). **(C)** *KLHL35* (chr11:75,128,618-75,144,419). Tracks for RNA-seq, DNA methylation, and chromatin state segmentation are shown. Chromatin segmentation profiles are not available for adrenal gland or testis in the UCSC browser. Tracks and abbreviations are as in previous figures with the addition of Brain FG, frontal gyrus; Neurons, neuronal fraction from adult brain.

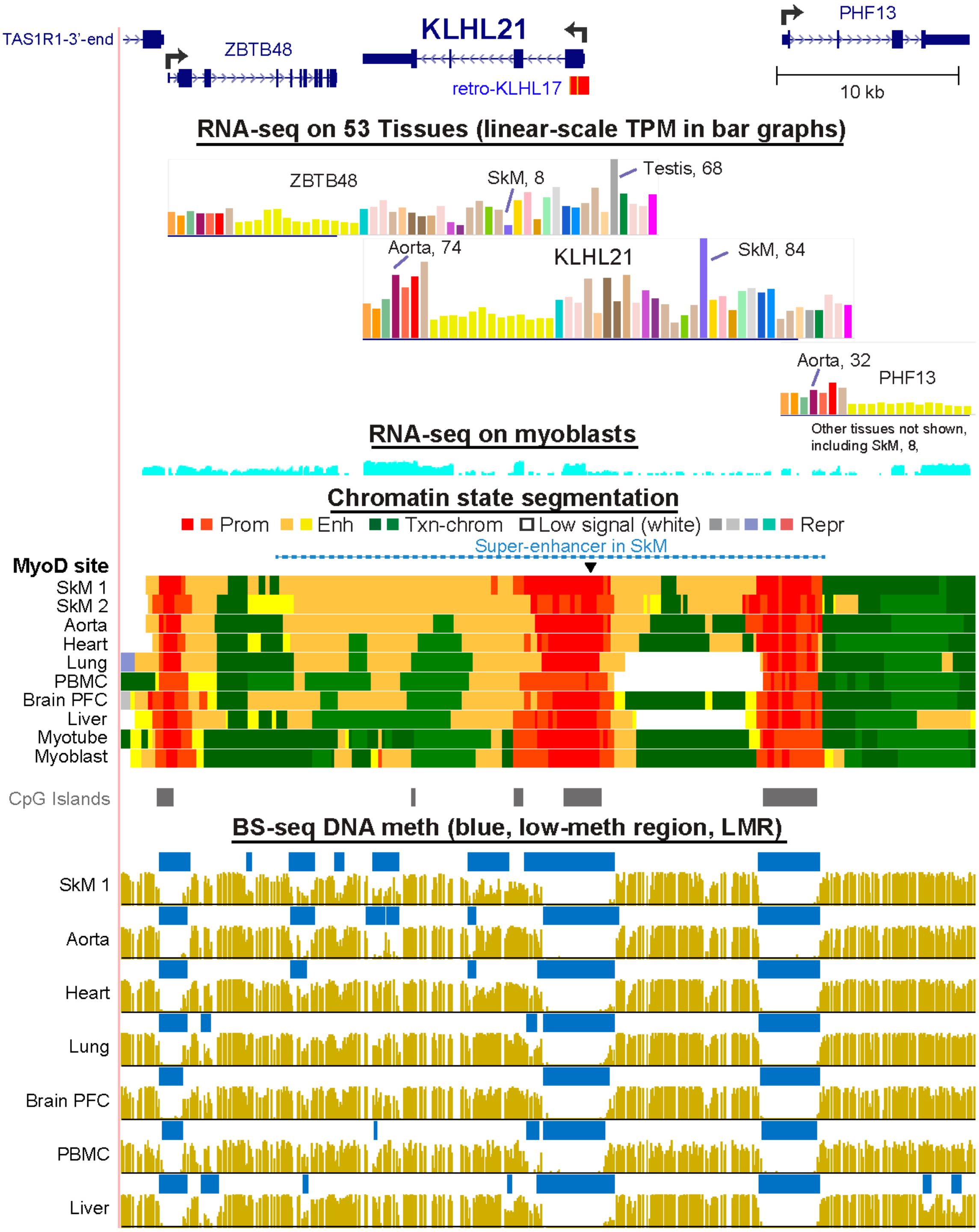

Figure S10. ***KLHL21*****, a gene required for cytokinesis, is widely expressed although with highest expression in skeletal muscle and has the most DNA hypomethylation and enhancer chromatin in skeletal muscle.** The region shown is chr11:75,128,618-75,144,419. Tracks are as previously described. Dotted line, a super-enhancer seen in skeletal muscle.
